# Fscn1 is required for the trafficking of TGF-β family type I receptors during endoderm formation

**DOI:** 10.1038/ncomms12603

**Published:** 2016-08-22

**Authors:** Zhaoting Liu, Guozhu Ning, Ranran Xu, Yu Cao, Anming Meng, Qiang Wang

**Affiliations:** 1 State Key Laboratory of Membrane Biology, CAS Center for Excellence in Molecular Cell Science, Institute of Zoology, Chinese Academy of Sciences, Beijing 100101, China; 2 Tsinghua-Peking Center for Life Sciences, School of Life Sciences, Tsinghua University, Beijing 100084, China; 3 Savaid Medical School, University of Chinese Academy of Sciences, Beijing 100049, China

## Abstract

Microtubules function in TGF-β signalling by facilitating the cytoplasmic trafficking of internalized receptors and the nucleocytoplasmic shuttling of Smads. However, nothing is known about whether actin filaments are required for these processes. Here we report that zebrafish actin-bundling protein *fscn1a* is highly expressed in mesendodermal precursors and its expression is directly regulated by the TGF-β superfamily member Nodal. Knockdown or knockout of *fscn1a* leads to a reduction of Nodal signal transduction and endoderm formation in zebrafish embryos. Fscn1 specifically interacts with TGF-β family type I receptors, and its depletion disrupts the association between receptors and actin filaments and sequesters the internalized receptors into clathrin-coated vesicles. Therefore, Fscn1 acts as a molecular linker between TGF-β family type I receptors and the actin filaments to promote the trafficking of internalized receptors from clathrin-coated vesicles to early endosomes during zebrafish endoderm formation.

## Abstract (web summary)

It is unclear how the cytoskeleton acts to assist in TGF-β signalling downstream of the receptor. Here, in zebrafish, the authors show that the actin-bundling protein FSCN1 interacts with TGF-β type I receptors ALK 4 and 5, enabling actin filament mediated vesicle trafficking and endoderm formation.

## Graphical abstract

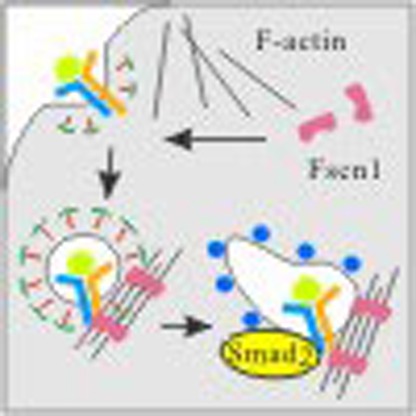

Members of the transforming growth factor-β (TGF-β) superfamily elicit intracellular signalling events by binding to and bringing together their cell surface type I and type II receptors, followed by type II receptor-mediated activation of type I receptors. The activated type I receptors subsequently phosphorylate downstream Smad effectors, then the phosphorylated Smads form heteromeric complexes with Smad4 and translocate into the nucleus to regulate target gene expression^1^. Since TGF-β superfamily members have been implicated in many important physiological processes, both the signalling duration and intensity are under tight cellular control.

TGF-β family receptors can be internalized via two major endocytic pathways, including clathrin-mediated endocytosis and lipid raft/caveolae-mediated endocytosis. Clathrin-mediated endocytosis facilitates TGF-β signalling in EEA1-positive early endosomes, where the Smad2 anchor for receptor activation (SARA) is enriched. Conversely, caveolae-mediated endocytosis attenuates signal activity by enhancing TGF-β type I receptor ubiquitination in caveolin-positive vesicles containing both the E3 ubiquitin ligase Smurf2 and the inhibitory molecule Smad7 (ref. 2). The regulated ubiquitination and degradation of internalized TGF-β family receptors have been proposed to play a crucial role in normal embryonic patterning^3^,^4^, but the importance of clathrin-mediated endocytosis of TGF-β family receptors in embryonic development has not yet been elucidated.

The dynamic polymerization of actin proteins not only has a central role in cell adhesion, migration and polarity, but also participates in endocytic internalization^5^. In yeast, genetic analyses have clearly shown that an intact actin cytoskeleton is required for the successful progression and execution of clathrin-mediated endocytosis^6^,^7^,^8^. In mammalian cells, however, actin only appears necessary when endocytosis occurs in locations that are dense with actin filaments^9^,^10^. Imaging of clathrin-coated structures in living cells has shown a close association between actin filaments and clathrin during endocytosis, suggesting that actin may provide an intracellular means for the formation and movement of endocytic vesicles^11^,^12^. As TGF-β signalling controls the actin cytoskeletal machinery to induce changes in cell shape and motility, a role for actin filaments in TGF-β receptor endocytosis has been suggested, but not yet firmly established^13^.

Fascin actin-bundling proteins (Fscns) cross-link filamentous actin into tightly packed parallel bundles and play a central role in architectural maintenance and functioning of cell protrusions^14^. Vertebrate genomes encode three forms of Fscn: Fscn1, Fscn2 and Fscn3. *Fscn1* is expressed in the nervous system and mesenchymal tissues while *Fscn2* and *Fscn3* are expressed in the retina and testes, respectively^15^. The developmental functions of Fscns have been investigated in various animal models. Female *Drosophila fscn1* mutants are sterile and display severe defects in bristle extension and blood cell migration^16^,^17^. Zebrafish maternal/zygotic *fscn1a* null mutants (MZ*fscn1a*) have severe loss of filopodia and a subset of neural crest cells display directional migration defects^18^. In contrast, *Fscn1*-deficient mice are viable and fertile with no major developmental defects. This difference between models is likely due to murine compensation by other actin-bundling proteins such as α-actinin^19^,^20^. As the formation of actin bundles mediated by actin-bundling proteins is required for clathrin-mediated endocytosis and membrane receptors internalization^21^,^22^,^23^, it is important to determine whether Fscn1 has a role in regulating TGF-β family receptor endocytosis during embryonic development.

In this study, we report that Nodal signalling, a TGF-β superfamily member, is both necessary and sufficient for the mesendodermal expression of *fscn1a*. Knocking down or knockout *fscn1a* disrupts Nodal signalling transduction and endoderm formation during the early development of zebrafish embryos. Importantly, the regulating role of Fscn1 proteins in TGF-β signal transduction is conserved in mammalian cells. Fscn1 specifically interacts with TGF-β type I receptor ALK5 or Activin/Nodal type I receptor ALK4, serving as a molecular linker between these type I receptors and the actin cytoskeleton to facilitate the trafficking of internalized receptors from clathrin-coated vesicles to early endosomes. Our findings indicate that *fscn1a* and Nodal signalling promote endoderm formation through a positive-feedback loop and may allow for a better understanding of how TGF-β signalling is elevated in Fscn1 overexpressed metastatic tumours.

## Results

### *fscn1a* is a Nodal target with mesendodermal expression

**Figure 1:**
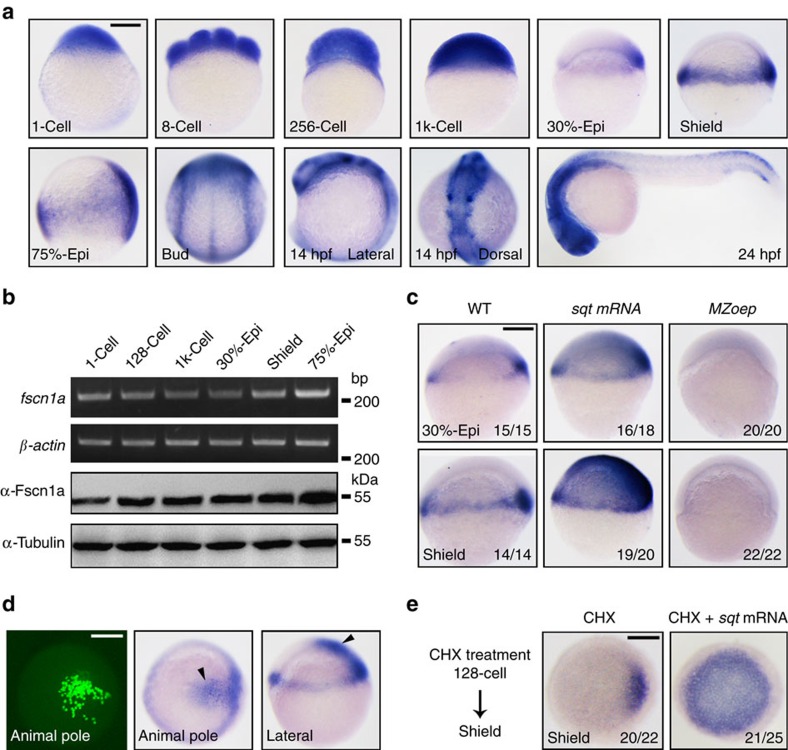
Zebrafish *fscn1a* is a direct TGF-β/Nodal target gene. (**a**) The spatiotemporal expression pattern of *fscn1a* during zebrafish embryogenesis was examined by *in situ* hybridization at the indicated stages. Stages and directionality are as follows: From the 1-cell to 1k-cell stages, lateral views with animal pole to the top; from 30% epiboly to mid-gastrulation stages, lateral views with dorsal to the right; bud stage, dorsal view with anterior to the top; 24 h.p.f., lateral views with anterior to the left. epi, epiboly. (**b**) Temporal expression profile of *fscn1a* was determined by semi-quantitative RT–PCR (upper panels) and western blot (lower panels). Total RNA isolates and proteins were prepared from embryos at the indicated stages. (**c**) *fscn1a* expression was assessed by *in situ* hybridization in wild-type (WT) and MZ*oep* mutant embryos. Embryos injected with 1 pg *sqt* mRNA were also collected at the 30% epiboly and shield stages and subjected to *in situ* hybridization. The ratios of affected embryos are indicated. (**d**) 0.05 pg *sqt* mRNA was injected into a single cell located in the animal pole at the 64-cell stage. 20 pg *egfp* mRNA was co-injected as a cell lineage tracer. Left panel, EGFP-labelled cells were observed at the shield stage by fluorescence microscopy; middle and right panels, *in situ* hybridization analysis of *fscn1a* expression in shield stage embryos following single-cell injection. (**e**) *fscn1a* expression in *sqt* mRNA injected embryos after CHX treatment. Embryos were injected with 1 pg *sqt* mRNA at the one-cell stage and incubated with 50 μg ml^−l^ CHX from the 128-cell to shield stages and then collected for *in situ* hybridization. Animal pole views with dorsal to the right. Scale bar, 200 μm.

Nodal/Smad2 signalling is essential for the formation and patterning of mesoderm and endoderm during vertebrate embryonic development^24^,^25^. We previously identified a number of Nodal/Smad2 direct targets including *fscn1a* encoding a key actin-bundling protein that is overexpressed in most carcinomas^26^. Due to whole-genome duplication, zebrafish has two *fscn1* orthologues named *fscn1a* and *fscn1b*. To explore which *fscns* might function in zebrafish mesendoderm formation, we first examined their temporal expression by whole-mount *in situ* hybridization (WISH). The *fscn1b* transcript was not observed before and at 24 h post fertilization (h.p.f.), but expressed in specific neurons in the brain at 36 h.p.f. as previously reported (Supplementary Fig. 1)^18^. In contrast, as shown in Fig. 1a, the *fscn1a* transcript was ubiquitously present in embryos from the 1-cell to 1 k-cell stages, indicative of maternal origin. From 30% epiboly to the shield stage, the zygotic expression of *fscn1a* was restricted to the germ ring and dorsal blastoderm margin where mesendodermal precursors reside. With the separation of mesoderm and endoderm from mesendoderm during gastrulation, *fscn1a* appeared expressed in migrating endodermal cells (75% epiboly stage, Fig. 1a). In addition, as previously reported, the *fscn1a* transcript was predominantly distributed in neural crest cells at the end of the gastrulation and the segmentation stages^18^. At 24 h.p.f., *fscn1a* was mainly expressed in the head and spinal cord dorsal neurons (Fig. 1a). We also investigated *fscn1a* expression using semi-quantitative reverse transcription–PCR (RT–PCR) and western blots. These analyses provided further evidence for the presence of both *fscn1a* transcripts and proteins before and after the mid-blastula transition (Fig. 1b).

To verify whether *fscn1a* is a direct Nodal target gene during mesendoderm induction, we examined its expression in embryos that had either excessive or deficient Nodal signal. Indeed, the expression of *fscn1a* was markedly increased in the Nodal ligand *squint* (*sqt*) messenger RNA (mRNA) injected embryos, but almost absent in Nodal-deficient MZ*oep* mutants at the 30% epiboly and shield stages (Fig. 1c). We also generated small clones of *sqt* overexpressed cells within whole embryos by co-injecting *sqt* mRNA together with *egfp* mRNA into one blastomere of a 64-cell embryo. The embryos with EGFP-labelled descendant cells located in the animal pole at the shield stage were then selected for *fscn1a* expression analysis. *fscn1a* transcript was highly locally induced, indicating that Nodal signalling is sufficient to activate its transcription (Fig. 1d). More importantly, inhibition of protein synthesis by Cycloheximide (CHX) treatment did not block ectopic Nodal signal-induced *fscn1a* expression (Fig. 1e). Taken together, these results suggest that the mesendodermal expression of zygotic *fscn1a* is directly regulated by Nodal signalling.

### *fscn1a* is essential for endoderm formation

To investigate the developmental function of *fscn1a*, we designed and synthesized two antisense morpholinos (MO1 and MO2), which interfered with translation by targeting different sequences of *fscn1a* and efficiently blocked the production of the Fscn1a-GFP (green fluorescent protein) fusion protein in embryos (Supplementary Fig. 2a,b). The expression of endogenous Fscn1a protein was also obviously depressed in *fscn1a* morphants (Supplementary Fig. 2c). Recently, it was shown that the first neural crest stream fail to migrate to the pharyngeal arches in *fscn1a*-deficient zebrafish embryos^18^. We found no difference in neural crest induction between mis-MO1 and MO1 injected embryos at 11 h.p.f. (Supplementary Fig. 2d), but the number of *dlx2a*-expressing neural crest cells in the first stream was obviously decreased in *fscn1a* morphants at 18 h.p.f. suggesting the efficiency and specificity of these two MOs (Supplementary Fig. 2e).

**Figure 2:**
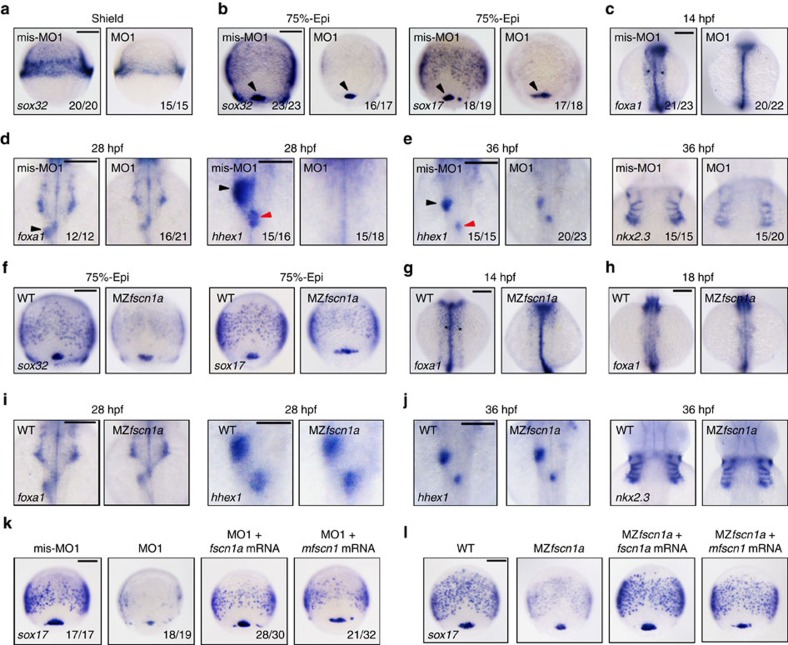
*fscn1a*-depletion disrupts endoderm formation. (**a**–**e**) The expression of endodermal markers in *fscn1a* morphants and control embryos at shield stage (**a**, Scale bar, 200 μm), 75% epiboly stage (**b**; Scale bar, 200 μm), 14 h.p.f. (**c**; Scale bar, 200 μm), 28 h.p.f. (**d**; Scale bar, 200 μm in the left two panels and 100 μm in the right two panels) and 36 h.p.f. (**e**; Scale bar, 200 μm). (**a**) Lateral views with dorsal to the right. (**b**–**e**) Dorsal views with anterior to the top. Dorsal forerunner cells (DFCs) were indicated by a black arrowhead (**b**) and the developing gut tube was indicated by black star (**c**). The liver and dorsal pancreatic buds were indicated by a black and a red arrowhead, respectively (**d**,**e**). Embryos in **a**, **b**, **c** and **d** were injected with 4 ng *fscn1a* mis-MO1 and MO1, while embryos in **e** were injected with 2 ng indicated MOs. (**f**–**j**) The expression of endodermal markers in MZ*fscn1a* mutants and wild-type embryos at 75% epiboly stage (**f**; Scale bar, 200 μm), 14 h.p.f. (**g**; Scale bar, 200 μm), 18 h.p.f. (**h**; Scale bar, 200 μm), 28 h.p.f. (**i**; Scale bar, 200 μm in the left two panels and 100 μm in the right two panels) and 36 h.p.f. (**j**, Scale bar, 200 μm). Note that the formation of the endodermal related tissues including the developing gut tube, the liver and pancreatic buds and the endoderm pouches was progressively recovered from 18 h.p.f. in MZ*fscn1a* mutants. (**k**,**l**) Ectopic expression of zebrafish *fscn1a* or mouse *fscn1* (*mfscn1*) in *fscn1a* morphants (**k**; Scale bar, 200 μm) or MZ*fscn1a* mutants (**l**; Scale bar, 200 μm) restores endoderm formation. Embryos were injected with 4 ng *fscn1a* MO1 alone or together with 150 pg *fscn1a* mRNA or *mfscn1* mRNA at the one-cell stage and collected at the 75% epiboly stage for *in situ* hybridization.

Consistent with the important function of *fscn1* in promoting cell migration, *fscn1a* morphants showed a slightly reduced epiboly progression (Supplementary Fig. 3a). Since *fscn1a* is specifically expressed in mesendodermal precursors, we investigated the expression of endodermal and mesodermal markers in *fscn1a* morphants. During gastrulation, injection of *fscn1a* MO1 drastically reduced the expression of endodermal marker *sox32* and *sox17* (refs 27, 28), while their expression in the dorsal forerunner cells was not affected (Fig. 2a,b). Likewise, injection of *fscn1a* MO2 similarly altered the expression patterns of *sox32* and *sox17*, suggesting target specificity (Supplementary Fig. 3b,c). Contrastingly, the expression of mesodermal markers including *gsc* and *ntl* was not noticeably changed (Supplementary Fig. 3d,e)^29^. To further confirm the function of *fscn1a* in endoderm formation, we examined the expression patterns of various endodermal tissue markers including *foxa1*, *hhex1* and *nkx2.3* (refs 30, 31, 32). We found that in *fscn1a* morphants, in comparison with control embryos, the expression of *foxa1* in the primitive gut tube at 14 h.p.f. was strikingly reduced (Fig. 2c). Furthermore, the liver and dorsal pancreatic buds were absent in *fscn1a* MO1 injected embryos as revealed by the expression of *foxa1* and *hhex1* at 28 h.p.f. (Fig. 2d). Injection of *fscn1a* MO1 to wild-type embryos resulted in obvious cell death in the head and a reduction in the size of the trunk at 24 h.p.f., but these morphological defects were largely disappeared in *p53* mutant embryos injected with *fscn1a* MO1 (Supplementary Fig. 4a), suggesting *fscn1a* MO1 injection could induce nonspecific *p53* activation. Injection MO1 into *p53* mutants led to similar impaired endoderm development suggesting that the endoderm defects in *fscn1a* morphants were not caused by morpholino-induced *p53* activation (Supplementary Fig. 4b,c). As these 4 ng MO1 injected wild-type embryos start to die after 28 h.p.f., we reduced the injection amount of MO1 to 2 ng and found that the development of the pancreatic bud was almost normal, but the formation of the liver bud and endoderm pouches were severe disrupted in morphants at 36 h.p.f. (Fig. 2e). These results suggest that the failure of endoderm formation in *fscn1a* morphants is not merely a transient intermediate, but also affects the development of organs derived from the endoderm.

To clarify whether the reduced number of endodermal precursor cells in our morphants was specific to the depletion of *fscn1a*, we further knocked out the *fscn1a* gene by transcription activator-like effector nucleases technology and reexamined the expression patterns of endodermal markers^33^,^34^. By targeting exon 1 of *fscn1a*, we obtained one mutation of (7 bp deletion with 1 bp insertion), which led to a shift of the open reading frame with a premature stop codon (Supplementary Fig. 5a). Since maternal Fscn1a protein masks the roles of zebrafish zygotic *fscn1a* (ref. 18), we further generated MZ*fscn1a* mutant by incrossing homozygous *fscn1a* zygotic mutants. In comparison with the wild-type control, the expression of *fscn1a* transcripts, except for in the forerunner cells, was totally abolished in MZ*fscn1a* mutant embryos (Supplementary Fig. 5b). As expected, Fscn1a protein was absent in MZ*fscn1a* mutants revealed by western blotting experiments (Supplementary Fig. 5c). Our MZ*fscn1a* mutants showed no obvious defects in neural crest induction, but displayed migration imperfections of the *dlx2a*-expressing neural crest cells (Supplementary Fig. 5d). These results confirm that the interrupted gene in our MZ*fscn1a* mutant line is *fscn1a*. Interestingly, the converging endodermal sheets were clearly missing in MZ*fscn1a* mutants during and after gastrulation (Fig. 2f,g), while the formation of the endodermal related tissues including the developing gut tube, the liver and pancreatic buds and the endoderm pouches was progressively recovered in MZ*fscn1a* mutants, suggesting that there could be some unknown mechanisms to compensate the function of *fscn1a* at later stages (Fig. 2h–j). In addition, MZ*fscn1a* mutants also showed retarded epiboly progression, but normal mesodermal germ layer formation (Supplementary Fig. 6a,b).

Off-target effects are of great concern with MO-based gene knockdown or transcription activator-like effector nuclease-based knockout applications. We conducted morpholino injection experiments in MZ*fscn1a* mutants to address this issue. Like *fscn1a* morphants, MZ*fscn1a* mutant embryos injected with *fscn1a* MO1 also exhibited obvious head necrosis, which was eliminated by coinjection of *p53* MO, indicating the nonspecific toxicity of *fscn1a* MO1 (Supplementary Fig. 6c). On the other hand, as revealed by *sox17* expression, there was no visible enhancement of endoderm defects in MO1 injected MZ*fscn1a* embryos compared with uninjected mutants (Supplementary Fig. 6d). Likewise, the liver and pancreatic buds were absent in *fscn1a* morphants at 28 h.p.f., but normally formed in MZ*fscn1a* mutants injected with or without *fscn1a* MO1 (Supplementary Fig. 6e). These results further demonstrate the specificity of the MOs we used in the current study. We also synthesized capped zebrafish *fscn1a* and mouse *fscn1* (*mfscn1*) mRNAs for rescue experiments. Injection of *fscn1a* or *mfscn1*mRNA restored the expression of *sox17* in *fscn1a* morphants and MZ*fscn1a* mutants (Fig. 2k,l). Taken together, these results show that *fscn1a* is crucial for the formation of the endodermal germ layer during zebrafish embryonic development.

### Fscn1 plays a positive role in Nodal signal transduction

**Figure 3:**
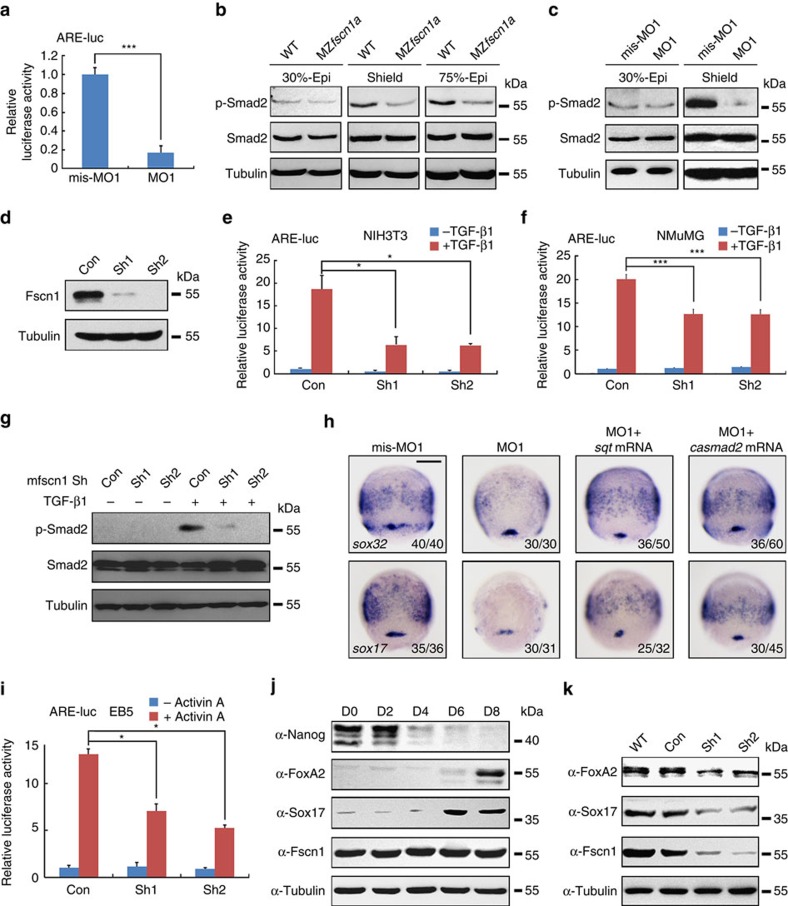
*fscn1a*-depletion inhibits Nodal signalling. (**a**) Reduction of ARE-luciferase reporter expression in *fscn1a* morphants. Embryos were co-injected with the reporter plasmids and mis-MO1 or MO1 at the one-cell stage and collected at the 75% epiboly stage for luciferase activity analysis. Data presented as mean with s.d. Student's *t* test, *n*=3, ***P<0.001. (**b**,**c**) Wild-type and MZ*fscn1a* mutant embryos (**b**) and embryos injected with mis-MO1 or MO1 (**c**) were collected at indicated stages for immunoblotting. (**d**) The effectiveness of mouse fscn1 shRNAs. NMuMG cells were transfected with 8 μg indicated shRNA plasmids per 100 mm dish and collected 48 h after transfection for western blot analyses. (**e**–**g**) NIH3T3 and NMuMG cells transfected with indicated shRNA plasmids (2 μg shRNA plasmids together with or without 0.5 μg reporter plasmids in one well of six-well plate) were treated with TGF-β1 (5 ng ml^−l^) for 12 h (**e**,**f**) or 3 h (**g**), and collected for luciferase measurements (**e**,**f**) or immunoblotting (**g**). The relative luciferase activity was the mean with s.d. from three independent biological repeats. Student's *t* test, *P<0.05, ***P<0.001. (**h**) Overexpression of *sqt* or *casmad2* rescues endoderm induction in *fscn1a* morphants. Embryos were injected with indicated MOs or mRNAs at the one-cell stage and collected at the 75% epiboly stage for *in situ* hybridization with *sox32* and *sox17* probes. Injection doses: mis-MO1, 4 ng; MO1, 4 ng; *sqt* mRNA, 1 pg; *casmad2* mRNA, 100 pg. Scale bar, 200 μm. (**i**) EB5 cells transfected with ARE-luciferase reporter together shRNA plasmids and treated with or without 25 ng ml^−1^ Activin A for 12 h before collected for luciferase assays. Data presented as mean with s.d. Student's *t* test, *n*=3, *P<0.05. (**j**) Western blots for the expression of Nanog, FoxA2, Sox17 and Fscn1 in samples taken at specified time points during embryoid body formation. The expression of Tubulin was examined as loading control. (**k**) Fscn1 knockdown disturbs the formation of endodermal cell lineage during embryoid body differentiation. Embryoid bodies on day 8, derived from EB5 cells infected with the indicated lentiviral shRNAs, were collected for western blots with indicated antibodies.

To clarify the function of Fscn1a in Nodal signalling, we first examined the effect of *fscn1a* knockdown in embryos on the expression of an ARE-luciferase reporter, which was driven by the Activin responsive elements, and found that the luciferase activity of this reporter was decreased by approximately fivefold in MO1 injected embryos (Fig. 3a). Interestingly, the expression level of p-Smad2 was not changed in MZ*fscn1a* mutants or morphants at 30% epiboly stage, whereas drastically decreased on *fscn1a* depletion at and after shield stages (Fig. 3b,c). These results indicate that *fscn1a* is necessary for Nodal signalling in the zebrafish embryo.

We extended these observations to mammalian cells via *Fscn1* depletion using two lentivirus vectors expressing short hairpin RNAs (shRNAs, named as Sh1 and Sh2) against two different sequences of the mouse *Fscn1*. Both shRNAs effectively knocked down endogenous Fscn1 protein expression in NMuMG cells (Fig. 3d). Furthermore, TGF-β1 treatment strongly activated ARE-luciferase expression in both NIH3T3 and NMuMG cells, whereas shRNA-mediated knockdown of *Fscn1* clearly reduced TGF-β1-induced luciferase activity (Fig. 3e,f). The TGF-β1-induced p-Smad2 level was much lower in NMuMG cells expressing either Sh1 or Sh2 than in controls expressing a scrambled control shRNA and knockdown with Sh2 exhibited a greater effect (Fig. 3g). In addition, knockdown of Fscn1 in NIH3T3 and NMuMG cells also impaired Activin A-induced ARE-luciferase expression (Supplementary Fig. 7a and b). Therefore, like the zebrafish Fscn1a, mammalian Fscn1 is required for TGF-β/Activin signal transduction. Importantly, co-injection of either *sqt* mRNA or constitutively active *smad2* (*casmad2*) mRNA, encoding zebrafish Smad2 mutant carrying two mutant amino acids corresponding to S465E/S467E of mammalian orthologues^35^, rescued the profound defects of endoderm formation in *fscn1a* morphants (Fig. 3h). This result suggests that *fscn1a* is a positive regulator of Nodal signalling during endoderm formation.

Although the mesodermal germ layer formation was not disrupted in *fscn1a* morphants (Supplementary Fig. 3d,e), we speculated that *fscn1a* might function in *sqt* or *casmad2* ectopic expression-induced mesoderm formation. As expected, injection of *fscn1a* MO1 into wild-type embryos obviously reduced the expression of endodermal markers compared with uninjected control, but did not changed the expression of *gsc*, *flh*, *efnb2b* and *pitx2a*, all of which are expressed in mesoderm and previously identified as Nodal/Smad2 direct target genes (Supplementary Fig. 7c–e)^26^,^36^, suggesting the mesodermal germ layer formation was not disrupted in *fscn1a* morphants. When injected with 0.5 pg *sqt* mRNA, embryos showed markedly induced expression of both mesodermal and endodermal markers, which was evidently reduced by co-injection 4 ng MO1 (Supplementary Fig. 7c–e). Thus, these data suggest that *fscn1a* is essential for Nodal signal to regulate target gene expression. However, co-injection of MO1 into embryos had no effect on the *casmad2*-induced expression of these target genes (Supplementary Fig. 7c–e), indicating that Fscn1a acts upstream of Smad2 in the Nodal pathway.

To further explore the role of Fscn1 in mammalian cell differentiation by modulating Activin/Nodal pathways, we knocked down Fscn1 in EB5 mouse embryonic stem cells. As shown in Fig. 3i, Sh1- or Sh2-mediated depletion of Fscn1 in EB5 cells decreased Activin A-induced ARE-luciferase reporter expression. We next performed embryoid body experiments to examine endodermal differentiation in Fscn1-depleted EB5 cells. During 8 days of embryoid body culture, the expression of the pluripotent marker Nanog was gradually decreased, whereas the endodermal markers such as FoxA2 and Sox17 were obviously promoted in day 6 or day 8, indicating embryonic stem cells within embryoid bodies undergo efficient differentiation (Fig. 3j). However, when Fscn1 was depleted, the formation of endoderm was clearly disturbed (Fig. 3k). These results show that, like zebrafish Fscn1a, mouse Fscn1 plays a conserved role in the differentiation of embryonic stem cells into endodermal cell lineage through regulating Activin/Nodal signalling.

### Actin-bundling activity of Fscn1 promotes endoderm formation

**Figure 4:**
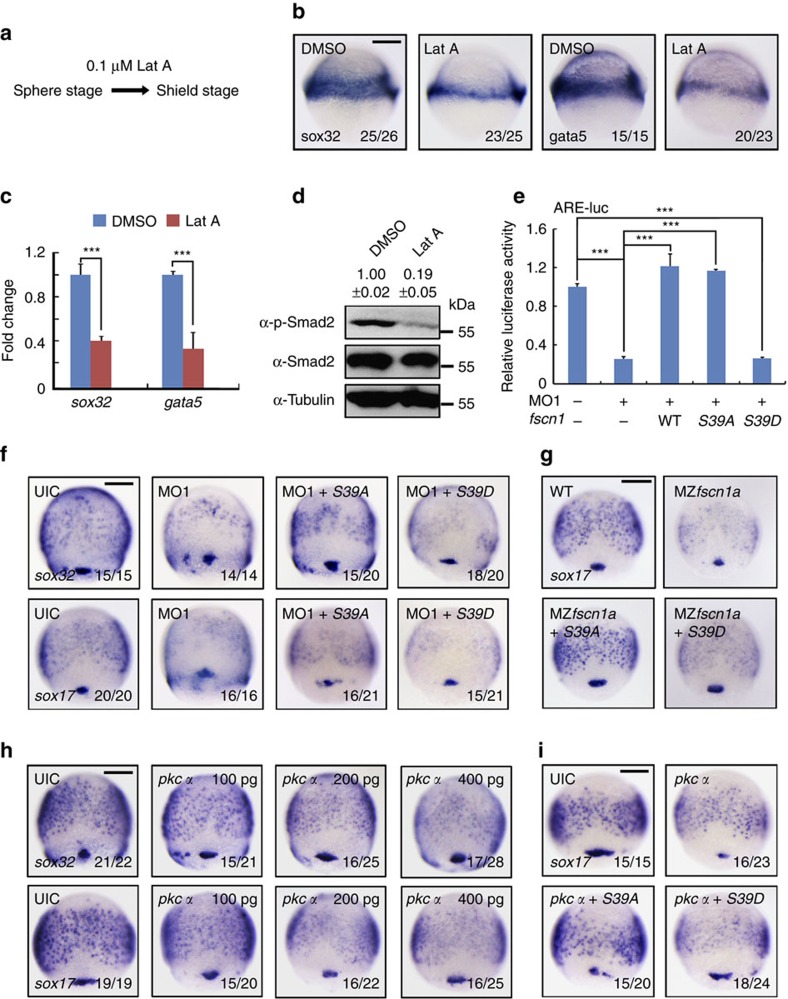
*fscn1* regulates endoderm formation and Nodal signal transduction via its actin-bundling activity. (**a**–**c**) The expression of endoderm markers *sox32* and *gata5* in wild-type embryos and embryos treated with Lat A. Embryos were treated with 0.1 μM Lat A from the sphere stage to shield stage (**a**), and analysed by *in situ* hybridization (**b**) and real-time PCR (**c**) for *sox32* and *gata5* expression. In **c**, the expression of *β-actin* was used as a reference to normalize the amount of mRNAs in each sample. The data are presented as mean ±s.d. of three independent experiments. Student's *t* test, ***P<0.001. (**d**) Actin filaments regulate Nodal/Smad2 signal transduction. Wild-type embryos were treated with 0.1 μM Lat A from the sphere to shield stages and then harvested for immunoblotting. Quantification is the relative density of phospho-specific signals to corresponding total protein signals (mean±s.d., three independent biological repeats). (**e**–**g**) Overexpression of *Fscn1 S39A*, but not *S39D*, recovers the expression reduction of ARE-luciferase reporters and endodermal markers in *fscn1a* morphants and MZ*fscn1a* mutants. Embryos were co-injected with the indicated MOs, plasmids and mRNAs at the one-cell stages and collected at the 75% epiboly stage for luciferase measurement (**e**) and *in situ* hybridization (**f**,**g**). The relative luciferase activity was the mean with s.d. from three independent experiments. Injection doses: ARE-luc, 100 pg; MO1, 4 ng; wild-type *fscn1* mRNA, 150 pg; *S39A* mRNA, 150 pg; *S39D* mRNA, 150 pg. Student's *t* test, ***P<0.001. (**h**) The expression of *sox32* and *sox17* were examined at the 75% epiboly stage in embryos injected with different dose of *pkcα* mRNA. (**i**) *Fscn1 S39A* overexpression antagonizes the PKCα-induced decrease of endoderm formation. One-cell stage embryos were co-injected with 400 pg *pkcα* mRNA and 150 pg *Fscn1 S39A* or *S39D* mRNA, then collected at the 75% epiboly stage for *in situ* hybridization and quantification of endodermal marker *sox17* expression. Scale bar, 200 μm.

Fscn1 is an actin-bundling protein that cross-links actin filaments into tightly packed, parallel bundles. We first investigated the implication of actin polymerization in endoderm formation. Embryos at the sphere stage were treated with Lat A, a cytoskeletal inhibitor that binds to monomeric actin and disrupts polymerization^37^, and harvested at the shield stage for examining the defects in endoderm formation (Fig. 4a). Results showed that Lat A treatment suppressed the expression of endoderm markers (*sox32* and *gata5*; Fig. 4b,c), but had no effects on the expression of the markers indicating mesoderm (*ntl*, *gsc* and *eve1*), non-neural ectoderm (*gata2*) and neuroectoderm (*otx2*; Supplementary Fig. 8)^38^, suggesting that actin skeleton plays a specific role in zebrafish endoderm formation. In addition, the p-Smad2 level was also obviously decreased in embryos treated with Lat A (Fig. 4d). Then, it is likely that actin filaments regulate endoderm formation by participating in Nodal signal transduction.

PKCα binds to and phosphorylates Fscn1 at serine 39 within the N-terminal actin-binding domain, resulting in the loss of actin bundles^39^,^40^. The constitutively active Fscn1 mutant S39A increased the number and length of filopodia, but the inactive Fscn1 mutants S39E or S39D did not, presumably due to their inability to bind to actin filaments^40^,^41^. On the basis of those prior works, we generated mouse *fscn1* point mutants *S39A* and *S39D* to examine the role of its actin-bundling activity in Nodal signal transduction and endoderm formation. The reporter activity of ARE-luciferase in MO1 injected embryos was de-repressed by co-injection 150 pg wild-type *fscn1* or *S39A* mutant mRNA, but not *S39D* mRNA (Fig. 4e). In agreement with these results, we found that the reduction of the endoderm markers *sox32* and *sox17* in *fscn1a* morphants and MZ*fscn1a* mutants could be largely recovered by overexpression of *S39A*, but not *S39D* (Fig. 4f,g). Furthermore, overexpression of PKCα in zebrafish embryos decreased *sox32* and *sox17* expression in a dose-dependent manner (Fig. 4h). Co-injection of mouse *fscn1* mutant *S39A* mRNA, but not *S39D* mRNA, effectively rescued the loss of endodermal fate in zebrafish embryos that had been injected with *pkcα* mRNA (Fig. 4i). Thus, these data suggest that the actin-bundling activity of Fscn1 is important for endoderm formation and Nodal signal transduction.

### Fscn1 interacts with TGF-β family type I receptors

**Figure 5:**
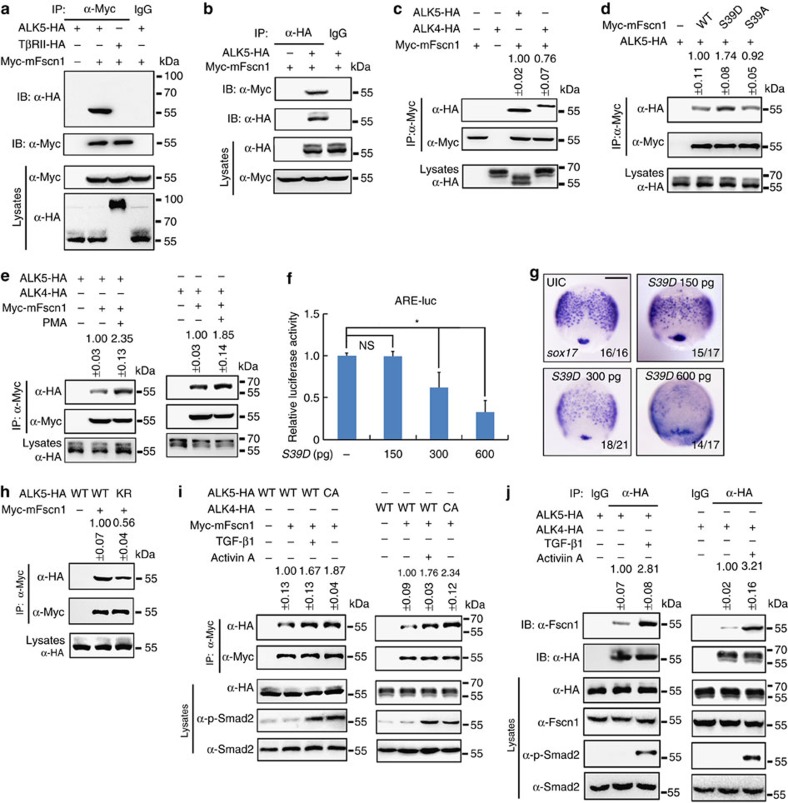
Fscn1 interacts with TGF-β family type I receptors. (**a**,**b**) Fscn1 interacts with TGF-β type I receptor ALK5. HEK293T cells were transfected as indicated with expression plasmids encoding Myc-tagged Fscn1 and HA-tagged- ALK5 or TGF-β type II receptor (TβRII) and collected for immunoprecipitation with anti-Myc (**a**) or anti-HA antibodies (**b**). (**c**) Fscn1 also associates with Nodal type I receptor ALK4. HEK293T cells transfected with the indicated constructs were subjected to immunoprecipitation. (**d**) Fscn1 S39D shows a stronger binding affinity for TGF-β family type I receptors. (**e**) PMA treatment elevates the receptor binding properties of Fscn1. HEK293T cells transfected with the indicated constructs were treated with 100 nM PMA for 2 h before collected for immunoprecipitation. (**f**,**g**) Embryos were co-injected with indicated doses of *S39D* mRNA at the one-cell stage and collected at the 75% epiboly stage for luciferase activity analysis (**f**) or *in situ* hybridization (**g**). Data presented as mean with s.d. Student's *t*-test, *n*=3, *P<0.05. NS, non-significant. UIC, uninjected control. Scale bar, 200 μm. (**h**) The receptor kinase activity of ALK5 is required for its binding to Fscn1. ALK5 K232R is a kinase-defective mutant carrying a lysine to arginine substitution in the putative ATP-binding site. (**i**) Fscn1 has a higher affinity for ligand-activated type I receptors. HEK293 cells transfected with the indicated constructs were treated with or without 5 ng ml^−l^ TGF-β1 or 25 ng ml^−1^ Activin A for 2 h before collecting for immunoprecipitation analysis. (**j**) HA-tagged ALK5 or ALK4 interacts with endogenous Fscn1. Note that the association of endogenous Fscn1 with HA-tagged ALK5 or ALK4 was enhanced in the presence of indicated ligands. The representative results of immunoprecipitation analysis were shown in **c**–**e** and **h**–**j**. Quantification is the mean relative ratio of co-immunoprecipitated signal over lysate (mean±s.d., at least three independent biological repeats). In all the immunoprecipitation analysis, HEK293T or HEK293 cells were cultured in 60 mm dishes. The dose of transfected plasmid DNA: ALK5-HA, 2 μg; CA-ALK5-HA, 2 μg; ALK5 KR-HA, 2 μg; ALK4-HA, 2 μg; CA-ALK4-HA, 2 μg; TβRII-HA, 1 μg; Myc-mFscn1, 1.5 μg; Myc-mFscn1 S39A, 1.5 μg; Myc-mFscn1 S39D, 1.5 μg.

Since Fscn1 acts upstream of Smad2 and its actin-bundling activity is required for Nodal signalling transduction (Supplementary Fig. 7c–e; Fig. 4e), we speculate that Fscn1 effects on the receptor level. To address this hypothesis, we first examined whether Fscn1 physically associates with TGF-β family receptors. Co-immunoprecipitation experiments revealed that Myc-Fscn1 was bound to TGF-β type I receptor ALK5, but not to type II receptor (Fig. 5a,b). The Activin/Nodal type I receptor ALK4, could also be detected in the Myc-Fscn1 complex with a relatively lower level (Fig. 5c). Interestingly, when compared with both wild-type and the S39A mutant, the Fscn1 S39D mutant had a stronger affinity for ALK5 (Fig. 5d). Furthermore, treatment of HEK293T cells with phorbol-12-myristate-13-acetate (PMA), a potent nanomolar activator of PKC, led to moderately elevated receptor-binding properties of Fscn1 (Fig. 5e). These results indicate that although the phosphorylated, inactive Fscn1 has lost its actin-bundling ability, it could more tightly associate with TGF-β family type I receptors. Furthermore, although injection 150 pg *S39D* mRNA into wild-type embryos had no detectable effects on Nodal signal transduction and endoderm formation, higher amount of injection (300 or 600 pg) led to evidently reduced Nodal signal activity and endoderm marker expression (Fig. 5f,g), showing the dominant negative effects of Fscn1 S39D as previously proposed^42^.

In addition, the amount of Myc-Fscn1 co-immunoprecipitated with HA tagged ALK5 K232R, a kinase-defective mutant carrying a lysine to arginine substitution in the putative ATP-binding site^43^, was decreased (Fig. 5h), indicating the requirement of the receptor kinase activity for binding to Fscn1. On the contrary, the association of overexpressed or endogenous Fscn1 with type I receptors (ALK5 and ALK4) was enhanced on TGF-β1 or Activin A stimulation (Fig. 5i,j), and a higher amount of Fscn1 proteins co-imunoprecipitated with the constitutively active form of ALK5 (caALK5 T204D) and ALK4 (caALK4 T206D), both of which were generated by replacing the indicated threonines of the GS domains (Fig. 5i)^44^,^45^. These results reveal that Fscn1 specifically interacts with TGF-β family type I receptors and has a higher affinity for the ligand-activated ALK5 and ALK4.

### Dynamin-dependent endocytosis facilitates endoderm formation

**Figure 6:**
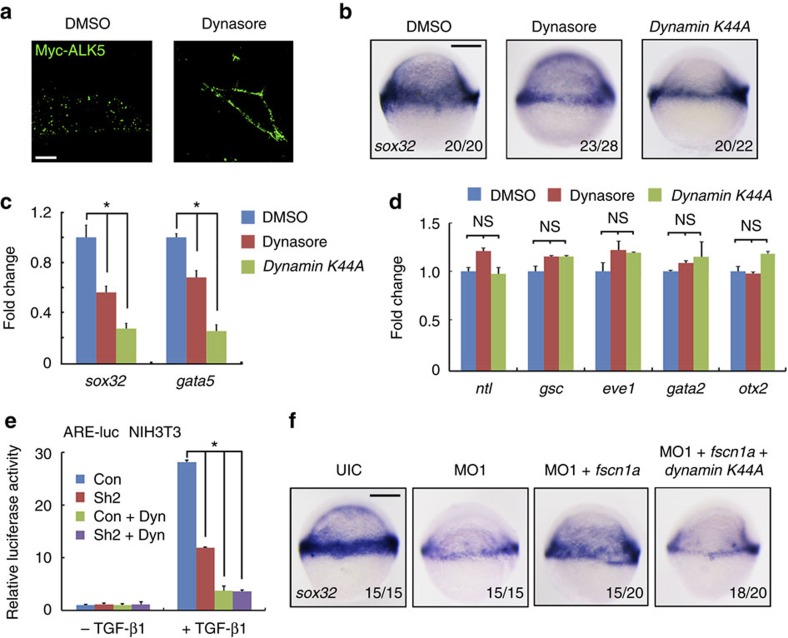
The functional connection between dynamin-dependent endocytosis and *fscn1a* during endoderm formation. (**a**) The inhibitory effect of dynasore on the endocytosis of membrane Myc-ALK5. NIH3T3 cells transfected with Myc-tagged ALK5 (1 μg in one well of six-well plate) were incubated with or without 80 μM dynasore for 30 min before antibody-labelled TGF-β type I receptor endocytosis assays. Then cells were incubated with anti-Myc antibody at 4 °C for 5 h, followed by incubation with PBS containing 10% knockout serum replacement (KSR) at 37 °C for 30 min. Dynasore were added during these incubation processes. The cells were visualized by immunofluorescence with anti-Myc (green) antibody. Scale bar, 5 μm. (**b**–**d**) The expression of *sox32* and *gata5* were assessed by *in situ* hybridization (**b**) and real-time PCR (**c**) at the shield stage in zebrafish embryos treated with 80 μM dynasore or injected with 400 pg of *dynamin K44A* mRNA. The expression of mesodermal (*ntl*, *gsc* and *eve1*), non-neural ectodermal (*gata2*) and neuroectodermal (*otx2*) markers were also examined by real-time PCR in these embryos (**d**). In **c** and **d**, the data are presented as mean±s.d. of three independent experiments. Student's *t*-test, *P<0.05. NS, non-significant. The expression of *β-actin* was used as a reference to normalize the amount of mRNAs in each sample. (**e**) NIH3T3 cells transfected with the indicated plasmids were treated with or without TGF-β1 (5 ng ml^−1^) and dynasore (80 μM) for 12 h, and then collected for luciferase measurements. The relative luciferase activity was the mean with SD from three independent experiments. Student's *t*-test, *P<0.05. (**f**) Inhibition of dynamin-dependent endocytosis impairs the *fscn1a* mRNA injection-induced rescue effects in *fscn1a* morphants. Embryos were co-injected with *fscn1a* MO1 and the indicated mRNAs at the one-cell stage and harvested at the shield stage for *sox32* detection.

To evaluate the importance of the dynamin-dependent endocytosis on zebrafish endoderm formation, we used dynasore, a chemical inhibitor of dynamin GTPase activity^46^, to inhibit embryonic dynamin-dependent endocytosis. As shown in Fig. 6a, most cell surface Myc-ALK5 proteins (ALK5 tagged with Myc right after signal peptide) were internalized into the cell cytoplasm in untreated NIH3T3 cells but hold up in the cell membrane in dynasore-treated cells, suggesting that dynasore treatment is capable of efficient inhibiting the dynamin-dependent endocytosis of TGF-β receptors. Next, dynasore treatment was applied to wild-type embryos. The results of WISH and quantitative RT–PCR (qRT–PCR) showed that the endoderm formation was clearly decreased in dynasore-treated embryos (Fig. 6b,c), whereas the induction of mesoderm and ectoderm was not disturbed (Fig. 6d). These observations were further confirmed by overexpressing dynamin using a dominant-negative mutant K44A to alternatively block dynamin-dependent endocytosis (Fig. 6b-d)^2^. Collectively, these data provide strong evidence for a functional link between dynamin-dependent endocytosis and endoderm development.

We then examined whether the function of Fscn1a in TGF-β signalling and endoderm formation is mediated by dynamin-dependent endocytosis. As shown in Fig. 6e, TGF-β1-induced ARE-luciferase reporter expression in dynasore-treated NIH3T3 cells was obviously suppressed compared with untreated control, but not further decreased by Fscn1-depletion. Importantly, the rescue effect of *fscn1a* mRNA for endoderm development in *fscn1a* morphants was completely reversed by co-injection of *dynamin K44A* mRNA into embryos (Fig. 6f). These results suggest that Fscn1 regulates TGF-β signal transduction and endoderm formation via a dynamin-dependent endocytosis pathway.

### Fscn1 mediates the subcellular trafficking of ALK5 and ALK4

**Figure 7:**
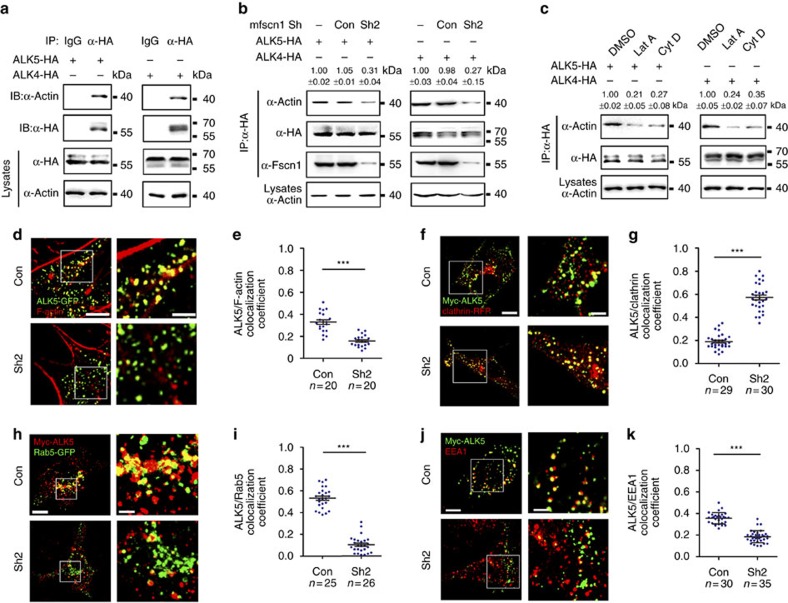
Downregulation of *Fscn1* sequesters the internalized receptors in clathrin-coated vesicles. (**a**) Actin interacts with TGF-β family type I receptors. NMuMG cells were transfected with 8 μg indicated constructs per 100 mm dish, and then subjected to immunoprecipitation. (**b**) Fscn1 mediates the interaction of actin with TGF-β family type I receptors. Note that Fscn1-deletion markedly decreased the association of actin and ALK5 or ALK4. (**c**) NMuMG cells expressing HA-tagged ALK5 or ALK4 were treated with 1 μM Lat A or 2 μM Cyt D for 3 h, then collected for immunoprecipitation. In **b** and **c**, quantification is the mean relative ratio of co-immunoprecipitated signal over lysate (mean±s.d., three independent biological repeats). (**d**,**e**) NIH3T3 cells cotransfected with indicated plasmids were fixed and costained with phalloidin-TRITC and anti-GFP antibody to show the co-localization of F-actin (red) and ALK5 (green). The right images (Scale bar, 2 μm) were amplified from the boxed areas in the corresponding left images (Scale bar, 5 μm) (**d**). Peason's co-localization coefficient was quantified from the indicated cell numbers in three independent experiments and the group values are expressed as mean±s.d. Student's *t*-test, ***P<0.001 (**e**). (**f**–**i**) NIH3T3 cells were transfected with indicated plasmids and then subjected to antibody-labelled receptor endocytosis. Scale bar, (**f**) left images, 5 μm; right images, 2 μm. Scale bar, (**h**) left images, 5 μm; right images, 1 μm. Peason's co-localization coefficient was quantified from the indicated cell numbers in three independent experiments and the group values are expressed as mean±s.d. Student's *t* test, ***P<0.001 (**g**,**i**). (**j**,**k**) NIH3T3 cells expressing Myc-ALK5 and the indicated shRNAs were subjected to antibody-labelled receptor endocytosis. Scale bar,: left images, 5 μm; right images, 2 μm. Peason's co-localization coefficient was quantified from the indicated cell numbers in three independent experiments and the group values are expressed as mean±s.d. Student's *t*-test, ***P<0.001 (**k**). In the co-localization analysis, NIH3T3 cells were cultured in six-well plates. The dose of transfected plasmid DNA: ALK5-GFP, 0.5 μg; Myc-ALK5, 1 μg; shRNA2, 2 μg; Clathrin-RFP, 0.5 μg; Rab5-GFP, 0.5 μg.

As several actin-binding proteins have been identified as molecular linkers between endocytic machinery and the actin cytoskeleton^47^,^48^,^49^, we hypothesized that Fscn1 serves as an adaptor connector for internalized TGF-β family type I receptors and the actin cytoskeleton during the process of dynamin-dependent endocytosis. To address this hypothesis, we first examined the interaction between actin and TGF-β family type I receptors in NMuMG cells. As shown in Fig. 7a, endogenous actin was efficiently co-immunoprecipitated by overpressed ALK5 and ALK4. However, Fscn1-deletion markedly decreased their association, suggesting that Fscn1 mediates the interaction of actin with TGF-β family type I receptors (Fig. 7b). Furthermore, when Lat A or Cytochalasin D (Cyt D), another inhibitor of actin polymerization^50^, was added to inhibit actin polymerization, the binding of actin to ALK5 and ALK4 were almost abolished, indicating that the interaction is between type I receptors and filamentous actin rather than monomeric actin (Fig. 7c). Likewise, we observed that abundant overexpressed ALK5 and ALK4 were co-localized with F-actin spots in NIH3T3 cells, but the calculated degree of association of these type I receptors and F-actin structures was decreased in Fscn1-depleted cells (Fig. 7d,e, Supplementary Fig. 9a,b). These results support the hypothesis that Fscn1 makes a bridge between internalized TGF-β family type I receptors and local F-actin structures.

We then explored whether Fscn1 deletion impaired the endocytosis of TGF-β family type I receptors in NIH3T3 cells. Surprisingly, Fscn1-deletion did not inhibit the internalization of the fixable analogue of FM4-64 (FM4-64FX) and membrane ALK5 (Supplementary Fig. 9c,d), indicating that Fscn1 is unnecessary for the formation and subsequent pinching off of endocytic vesicles from the plasma membrane. We next investigated whether Fscn1 regulates the localization of internalized type I receptors in clathrin-coated vesicles and early endosomes. In NIH3T3 cells transfected with scrambled shRNA, about 21% of internalized ALK5 was co-stained with clathrin-coated vesicles (Fig. 7f,g). However, in Fscn1-deficient cells, the proportion of ALK5-clathrin co-localization was markedly increased (Fig. 7f,g). Otherwise, the co-localization of internalized ALK5 and Rab5- or EEA1-positive early endosomes was obviously decreased in Fscn1-deficient cells (Fig. 7h-k).

**Figure 8:**
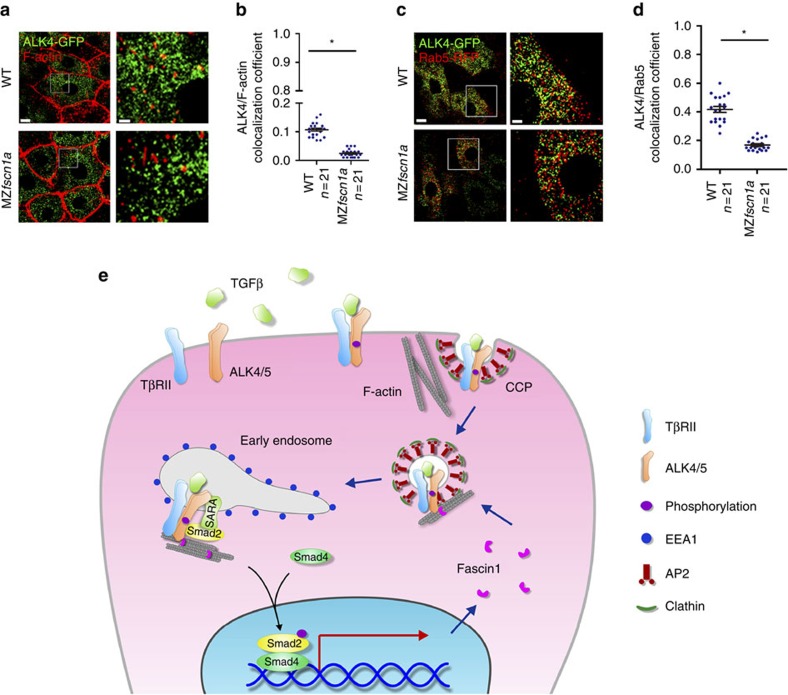
The endocytic trafficking defects of Activin/Nodal type I receptor ALK4 in MZ*fscn1a* mutants. (**a**,**b**) 20 pg ALK4-GFP mRNA was injected into one blastomere of a 16-cell wild-type or MZ*fscn1a* embryo. Embryos were harvested at shield stage and costained with phalloidin-TRITC and anti-GFP antibody to show the co-localization of F-actin (red) and ALK4 (green). GFP-positive hypoblast cells were selected and photoed using a Nikon A1R+ confocal microscope. The boxed area in the left image (Scale bar, 10 μm) is presented at a higher magnification in the corresponding right image (Scale bar, 2 μm) (**a**). Peason's co-localization coefficient was quantified from the indicated cell numbers in three independent experiments and the group values are expressed as mean±s.d. Student's *t*-test, *P<0.05 (**b**). (**c**) Immunostaining images of Rab5-RFP (red) and ALK4-GFP (green) in hypoblast cells of wild-type or MZ*fscn1a* embryos. 20 pg ALK4-GFP mRNA and 20 pg Rab5-RFP mRNA were co-injected into one blastomere of a 16-cell wild-type or MZ*fscn1a* embryo. Embryos were collected at shield stage for immunostaining with anti-GFP and anti-dsRed antibodies. Scale bar, left images, 10 μm; right images, 3 μm. (**d**) Peason's co-localization coefficient of ALK4-GFP and Rab5-RFP was quantified from the indicated cell numbers in three independent experiments and the group values are expressed as mean±s.d. *P<0.05. (**e**) Proposed mechanism of the function of Fscn1 in trafficking and signalling of TGF-β type I receptors. TGF-β/Smad signal induces the expression of *fscn1* gene and Fscn1 protein acting as a molecular linker between TGF-β family type I receptors and the actin cytoskeleton, which promotes the trafficking of internalized receptors from clathrin-coated vesicles to early endosomes, thereby enhancing TGF-β signal activity. TβRII, TGF-β type II receptor; CCP, clathrin-coated pit; AP2, assembly polypeptide 2.

Next, we further examined the endocytic trafficking defects of Activin/Nodal type I receptor ALK4 in MZ*fscn1a* mutants. Consistent with the results in mammalian cells, plenty of overexpressed ALK4 were co-localized with F-actin spots in the involuted hypoblast cells of wild-type embryos (Fig. 8a,b). From this result we could not deduced whether the Alk4/F-actin interaction occurs during receptor endocytosis or en route to the plasma membrane, but Fscn1 indeed acts as a molecular linker between ALK4 and the actin cytoskeleton as the co-localization of ALK4 and F-actin spots was evidently declined in MZ*fscn1a* mutants compared with wild-type control (Fig. 8a,b). Although the attempt to analyse the ALK4-clathrin co-localization in embryos was impeded by the lacking of effective zebrafish clathrin heavy chain antibody and the difficulty in synthesizing the corresponding mRNA of clathrin-RFP due to its large size (nearly 6,000 nucleotides), we indeed observed a sharp decline in the subcellular localization of ALK4 to early endosomes (Fig. 8c,d). Taking together, these results indicate that Fscn1 functions as a molecular linker between these internalized receptors and the actin cytoskeleton to facilitate the trafficking of TGF-β family type I receptors from clathrin-coated vesicles to early endosomes (Fig. 8e).

## Discussion

Zebrafish *fscn1a* is enriched in migrating neural crest cells and implicated in cell motility^18^. Our data indicate that zebrafish *fscn1a* is also highly and specifically expressed in mesendodermal cells. MO-based gene knockdown experiments reveal that *fscn1a* plays a vital role in the formation of the endodermal germ layer and related organs. Surprisingly, the MZ*fscn1a* mutants generated by our lab display severe defects of endoderm formation during and after gastrulation, but the development of the endodermal-related tissues are progressively recovered at later stages. Since the specificity of the MOs used to transient knockdown of *fscn1a* in embryos was verified by various alternative approaches, the different phenotypes observed between the MO-injected embryos and the related mutants might be resulted from the activation of a compensatory network to buffer against the deleterious mutation as recently reported^51^,^52^.

There is an abundance of literatures showing that the expression of *Fscn1* is regulated by TGF-β/Nodal signalling in various tumour cells^53^,^54^,^55^. In agreement with these previous findings, we reveal that Nodal signalling is both necessary and sufficient to activate *fscn1a* expression during zebrafish embryo gastrulation. Our previous work indicated that Smad2/4 could bind to the promoter of *fscn1a* (ref. 26). In a similar vein, recent work has revealed that TGF-β and Smad4 could induce *Fscn1* overexpression in basal-like breast cancer cells by directly binding to a Smad-binding element on the *Fscn1* promoter (ref. 56). Thus, the regulation of *Fscn1* expression by TGF-β signal is conserved across species. On the other hand, we found that knockdown or knockout of *fscn1a* markedly decreased the level of phosphorylated Smad2 in zebrafish embryos and mammalian cell lines. We therefore propose that *Fscn1* and Nodal signalling promote endoderm formation through a positive-feedback loop.

Interestingly, *fscn1a* morphants and MZ*fscn1a* mutants exhibit severe defects in endoderm formation, whereas the development of mesoderm cells is not affected. Many studies have revealed that high levels of Nodal signalling promote endoderm formation, while lower levels of Nodal signalling favour mesoderm differentiation^27^,^57^,^58^. Furthermore, later time points of Nodal signal blocking induce much weaker defects of mesoderm formation^59^. Since the activity of Nodal signal is not obviously changed before shield stage and not totally abolished during gastrulation in *fscn1a* morphants and MZ*fscn1a* mutants, it is understandable that the mesoderm is not obviously affected in *fscn1a*-depleted embryos.

The actin-bundling activity of Fscn1 is inhibited by phosphorylation at residue Ser-39 by PKC*α*, which abrogates the activity of the N-terminal actin-binding site^39^,^40^. Past work has indicated that PKCα^−/−^ podocytes showed a enhancement of TGF-β-induced Smad2-phosphorylation^60^, suggesting a possible regulatory role for PKCα-dependent phosphorylation of Fscn1 in TGF-β signal transduction. Overexpression of PKCα decreased endoderm formation through inactivation of the actin-bundling activity of endogenous Fscn1, supporting the idea that PKCα is involved in the mechanisms of *fscn1a*-regulated endoderm formation.

Clathrin-mediated endocytosis is a multi-step process for cargo internalization from the plasma membrane that entails endocytic vesicle formation, pinching off from the plasma membrane, intracellular trafficking, uncoating of the clathrin coat and selective fusion with endosomal and/or lysosomal compartments^61^,^62^. A recent study has elucidated that Fscn1 and another actin binding protein, VASP, synergistically increase the growth of the actin comet tail, which plays an important role in clathrin-mediated endocytosis^63^. However, we found that Fscn1 is dispensable for the formation and pinching off of endocytic vesicles, as indicated by our results showing that TGF-β tpe I receptors are normally internalized from the cell membrane to cytoplasm in Fscn1-depleted cells. Contrastingly, the internalized receptors are sequestrated in clathrin-coated vesicles and cannot be translocated into early endosomes when Fscn1 expression is inhibited. This suggests that Fscn1 is specifically required for the trafficking of TGF-β family type I receptors from clathrin-coated vesicles to early endosomes.

On the basis of our results, we propose a new working model for the functioning of Fscn1 during the formation of the endoderm in zebrafish embryos. The canonical TGF-β/Nodal signalling pathway in mesendodermal precursors induces the expression of *fscn1a*, with Fscn1 protein acting as a molecular linker between TGF-β family type I receptors and the actin cytoskeleton. This linkage facilitates the trafficking of the internalized receptors from clathrin-coated vesicles to early endosomes, thereby promoting TGF-β signal transduction and endoderm formation. Given the profound impacts of TGF-β signalling and Fscn1 on tumour cell migration and invasion^64^,^65^, it will be invaluable to examine whether the positive-feedback loop involving Fscn1 and TGF-β signal proposed here directs tumour metastasis.

## Methods

### Zebrafish strains

Wild-type embryos were obtained from natural matings of Tübingen zebrafish. Embryos were raised in Holtfreter's solution at 28.5 °C and staged by morphology as previously described^66^. MZ*oep* mutant embryos were generated by crossing homozygous male and female *oep*^*tz257/tz257*^ adult mutants, which were rescued by injection of *oep* mRNA. Homozygous *p53(M214K*) mutant embryos (abbreviated as *p53*^−/−^) carrying a loss-of-function *p53* point mutation were employed to exclude the contributions of the morpholino-induced non specific cell apoptosis. Our work involving zebrafish embryo collection and analysis was carried out in accordance with and approved through the Animal Care Committee at the Institute of Zoology, Chinese Academy of Sciences.

### Cell lines and transfections

HEK293, HEK293T, NMuMG and NIH3T3 cell lines were maintained in DMEM medium supplemented with 10% fetal bovine serum (FBS) in a 37 °C humidified incubator in a 5% CO_2_ environment. Transient transfections in these cell lines were performed using Lipofectamine 2000 (11668019, Invitrogen) following the manufacturer's instructions. Undifferentiated EB5 mouse embryonic stem cells, a subline generated by targeted integration of an Oct3/4-IRES-BSD-pA vector into the Oct3/4 allele and carries the blasticidin S-resistant selection marker gene driven by the Oct3/4 promoter, were maintained on gelatin-coated dishes in Glasgow minimum essential medium supplemented with 1% foetal calf serum, 10% knockout serum replacement, 0.1 mM nonessential amino acids, 1 mM sodium pyruvate, 0.1 mM 2-mercaptoethanol, 1,000 U ml^−l^ leukemia inhibitory factor (LIF) and 20 μg ml^−1^ blasticidin S.

### Plasmids and RNA interference

Zebrafish *fscn1a* and mouse *fscn1* were cloned into pcs2-Flag and pcs2-Myc vectors for eukaryotic expression. The plasmids pcs2-Myc-fscn1 S39A and S39D were constructed by the mutagenesis of pcs2-Myc-fscn1 using the Fast Mutagenesis System (FM111, TransGen Biotech) and confirmed by DNA sequencing. Rab5-GFP plasmid was bought from Origene. DynaminK44A plasmid was kindly gifted from Professor Ye-Guang Chen of Tsinghua University (Beijing, China). Professor. Xiaohong Fang of Institute of Chemistry, Chinese Academy of Sciences (Beijing, China) kindly provided the Clathrin-mRFP plasmid. For testing the effectiveness of *fscn1a* MO1 and MO2, the expression vector *fscn1a*-GFP was generated by fusing the 71 bp upstream flanking region and the first 276 bp of the *fscn1a* open reading frame into a pEGFP-N3 vector. For RNA interference, two *fscn1* shRNA constructs were generated using a pll3.7 plasmid. The targeted sequences were as follows: 5′- AACTCTTCCTCATGAAGC -3′ and 5′- AGGCTGTGCAGATTCAGT -3′.

### Formation of embryoid bodies

To induce the formation of embryoid bodies, EB5 cells infected with the indicated lentiviral shRNAs were treated with trypsin briefly to retain cells in medium sized clumps. Cells were then centrifuged at low speed and resuspended in ESC growth medium without LIF and cultured in bacteriological grade Petri dishes for embryoid body formation. Samples were taken at specified time points and subjected to western blots.

### RNA synthesis and WISH

Capped RNAs for zebrafish *fscn1a*, mouse *fscn1*, mouse *fscn1 S39A*, mouse *fscn1 S39D*, *sqt*, *constitutively active smad2 (casmad2)*, *dynamin K44A*, *pkcα*, ALK4-GFP and Rab5-RFP were synthesized *in vitro* from corresponding linearized plasmids using the mMessage mMachine kit (Ambion). Digoxigenin-UTP-labelled antisense RNA probes were transcribed using MEGAscript Kit (Ambion) according to the manufacturer's instructions. WISH with these RNA probes were performed using the NBT-BCIP substrate.

### Morpholinos

Two antisense morpholinos, *fscn1a* MO1 (5′- TGTCGCTGGTTCCGTTTGCAGTCAT -3′, positioning from 142 to 166 of *fscn1a* sequence, NM_001076560.1, NCBI) and *fscn1a* MO2 (5′- TTGCAGCTCGTGGCCTTGCTGGA -3′, positioning from 117 to 139 of *fscn1a* sequence, NM_001076560.1, NCBI) were designed. A control MO (mis-MO1) included six mismatched nucleotides from *fscn1a* MO1 and was designed as follows: 5′- TATCGAGGCATCCGTTTGCAGTCAT -3′. *p53* morpholino (5′- GCGCCATTGCTTTGCAAGAATTG -3′) was designed according to published sequence^67^. All of these MOs were synthesized by Gene Tools and resuspended in nuclease free water. MOs were microinjected into the fertilized eggs at the one-cell stage at indicated concentrations and cultured at 28.5 °C until use.

### Embryonic treatment

To block protein translation, wild-type embryos were treated with 50 μg ml^−l^ CHX (C7698, Sigma) at the 128-cell stage. Embryos were then collected at the shield stage for WISH. To disrupt dynamin-dependent endocytosis, embryos were treated with 80 μM dynasore (D7693, Sigma) at the 16-cell stage and collected at the shield stage for additional experimentation. To inhibit actin polymerization, embryos were treated with 0.1 μM Latrunculin A (Lat A, L5163, Sigma) from the sphere to shield stages and then subjected to either *in situ* hybridization or quantitative RT–PCR analysis.

### Dual-luciferase reporter assays

For detection and quantification of Smad2 activity in zebrafish embryos, the Smad2-specific ARE luciferase reporter construct DNA was mixed with Renilla luciferase reporter DNA in a ratio of 10:1. Wild-type embryos were co-injected with the aforementioned DNA mixture and either the indicated MOs or mRNAs at the one-cell stage. A group of 20 embryos from each sample were collected at the shield stage and lysed with passive lysis buffer to detect any luciferase activity.

For the cell culture luciferase reporter assays, either NIH3T3 or NMuMG cells were transfected with the indicated plasmids. The pRenilla-TK vector was used as an internal control. Cells were stimulated with 5 ng ml^−g^ TGF-β1 (240-B, R&D Systems) or 25 ng ml^−1^ Activin A (338-AC, R&D Systems) for 16 h before collecting for luciferase activity assays. Each luciferase reporter assay was performed in triplicate and the data represent the mean plus or minus s.d. of three independent biological repeats after normalized to Renilla activity.

### qRT–PCR

Total RNA was extracted with Trizol (Invitrogen) and complementary DNA was synthesized with high-efficiency reverse transcriptase Revertra Ace (Toyobo). A Biorad CFX96 PCR system was employed to perform qRT–PCR using SYBR Premix Ex Taq dye (Takara). The information of primer sequences were given in Supplementary Table 1.

### Antibodies and immunoprecipitation assays

For immunoblotting, we used the following affinity-purified antibodies: anti-Smad2/3 (1:1,000; 3102, Cell Signaling Technology), anti-p-Smad2 (1:500; AB3849, Millipore), anti-Smad3 (1:1,000; 9523, Cell Signaling Technology), anti-p-Smad3 (1:1,000; 04-1042, Millipore), anti-Fscn1 (1:5,000; 5535-1, Epitomics), anti-FoxA2 (1:1,000; 8186S, Cell Signaling Technology), anti-Nanog (1:1,000; Ab80892, Abcam), anti-Sox17 (1:1,000; 09038, Millipore), anti-Actin: (1:1,000; CW0096A, CWBIO), anti-Tubulin (1:1,000; CW0098A, CWBIO), anti-Myc (1:3,000; M20002, Abmart), anti-HA (1:3,000; M20003, Abmart) and anti-Flag (1:5,000; F3165, Sigma). Anti-Myc (1:100) and anti-HA (1:100) antibodies were also used in immunoprecipitation assays.

For each immunoprecipitation assay, either HEK293T or HEK293 cells were transfected with indicated plasmids. Cells were collected 48 h after transfection and lysed with TNE lysis buffer (10 mM Tris-HCl, pH 7.5, 150 mM NaCl, 2 mM EDTA, and 0.5% Nonidet P-40) containing a protease inhibitor cocktail. Lysates were incubated with protein A sepharose beads and indicated antibodies at 4 °C for 4 h. The beads were then washed four times with TNE buffer. The bound proteins were separated by SDS–PAGE and visualized by western blotting. The intensity of each band was quantified using Image J. Full images of uncropped western blots are shown in Supplementary Fig. 10.

### Antibody-labelled TGF-β type I receptor endocytosis

NIH3T3 cells were placed on glass coverslips and transfected with shRNA expressing plasmids against *Fscn1* and TGF-β type I receptor tagged with Myc right after signal peptide. Two days later, cells were washed with ice-cold PBS and then incubated with α-Myc antibody (1:100; M20002, Abmart) for 5 h at 4 °C. Then cells were washed with cold PBS and returned to 37 °C for 30 min, allowing endocytosis to occur. The cells were fixed and prepared for immunofluorescence analysis.

### Immunofluorescence

For immunofluorescence, cells were fixed in 4% paraformaldehyde and permeabilized for 10 min in PBS containing 0.2% Triton X-100. Cells were washed with PBS containing 0.2% Triton X-100 and 5% FBS, followed by incubation with anti-Myc (1:100; M20002, Abmart) or anti-dsRed (1:1,000; 632496, Clontech), anti-GFP (1:1,000; A11122, Invitrogen) or anti-EEA1 (1:100; 3288, Cell Signaling Technology) antibodies. Phalloidin-TRITC (p1951, Sigma) was used as an actin marker in related immunofluorescence assays.

For immunofluorescence assays in zebrafish embryos, indicated mRNAs were co-injected into one blastomere of a 16-cell wild-type or MZ*fscn1a* embryo. Embryos were collected at the shield stage, fixed in 4% paraformaldehyde overnight and then washed with PBS containing 0.1% Tween 20 for 30 min, blocked with 1% bovine serum albumin for 1 h at room temperature, followed by incubation with anti-dsRed and anti-GFP antibodies overnight.

Single *z*-plane images shown the internalized receptors in the cytoplasm were captured using a Nikon A1R+ confocal microscope. For quantitative co-localization analysis, Peason's Co-localization coefficient was measured using NIS-Elements AR analysis 4.13.00 64-bit software. All data are the mean of three independent biological repeats that gathered from more than 20 cells. All the group values are expressed as mean±s.d.

### FM4-64 staining

NIH3T3 cells were incubated with 4 μM FM4-64FX (1417661, Invitrogen) for 1 h. Excess dye was removed with a 10 min was in normal extracellular solution (136 mM NaCl, 2.5 mM KCl, 1.3 mM MgCl_2_, 10 mM Hepes, 2 mM CaCl_2_ and 10 mM glucose at pH 7.4). Cells were then fixed using 4% PFA for 10 min and washed in PBS containing 10% FBS for three times. The cells were imaged using a Nikon A1R+ confocal microscope.

### Statistical analyses

Student's *t*-test was used to analyse all data sets (Microsoft Excel software). At a minimum, experiments were performed in triplicate. Results were considered statistically significant at *P*<0.05.

### Data availability

The authors declare that the data supporting the findings of this study are available within the article and its Supplementary Information files or from the corresponding author on reasonable request.

## Additional information

**How to cite this article**: Liu, Z. *et al*. Fscn1 is required for the trafficking of TGF-β family type I receptors during endoderm formation. *Nat. Commun.* 7:12603 doi: 10.1038/ncomms12603 (2016).

## Supplementary Material

Supplementary Information Supplementary Figures 1-10
