# Supplementary material for: Fscn1 is required for the trafficking of TGF-β family type I receptors during endoderm formation
Source: Nat Commun. 2016 Aug 22;7:12603. doi: 10.1038/ncomms12603 (PMC4996939; doi:10.1038/ncomms12603)

Supplementary Figure 1

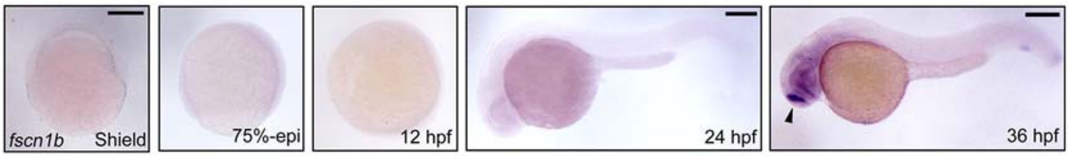

Supplementary Figure 1. The spatiotemporal expression pattern of *fscn1b* during zebrafish embryogenesis. The expression of *fscn1b* in wild-type embryos was examined by *in situ* hybridization at indicated stages. Shield and 75%-epiboly stages, lateral views with dorsal to the right; 12 hpf, dorsal view with anterior to the top; 24 and 36 hpf, lateral views with anterior to the left. The arrow head shows that *fscn1b* transcript was expressed in specific neurons in the brain. Scale bar, 200 μm.

Supplementary Figure 2

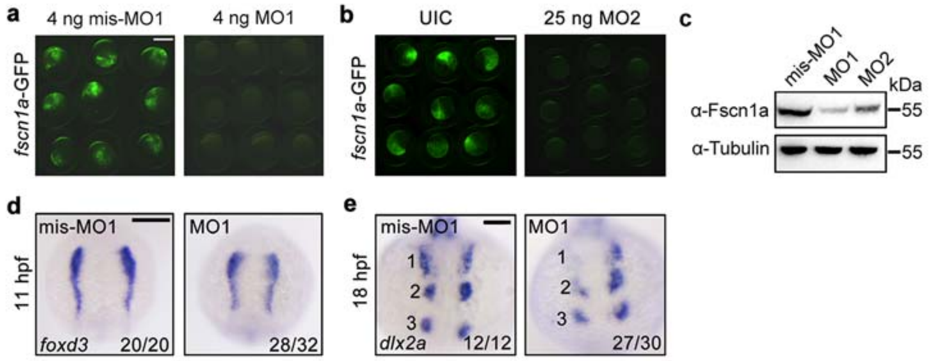

Supplementary Figure 2. *fscn1a* morphants exhibit NC cell migration defects in the first stream. (a-b) Effectiveness of *fscn1a* MO1 and MO2. Embryos co-injected with 50 pg *fscn1a*-GFP plasmid DNA and indicated MOs. Green fluorescence was detected at the shield stage. *fscn1a* MO1 (a) or MO2 (b) injected embryos showed a noticeable decrease in green fluorescence when compared to control embryos. Scale bar, 400 μm. (c) The expression of Fscn1a protein was examined by Western blots in shield-stage embryos injected with *fscn1a* MO1 (4 ng) or MO2 (25 ng). The expression of Tubulin was detected as loading control. (d-e) Embryos injected with mis-MO1 (4 ng) or *fscn1a* MO1 (4 ng) at the one-cell stage and harvested at indicated stages for *in situ* hybridization with *foxd3* (d) and *dlx2a* (e) probes. Dorsal views with anterior to the top. Numbers were labeled to show the three streams of pharyngeal NC cells. Scale bar, 200 μm in panel d and 100 μm in panel e.

Supplementary Figure 3

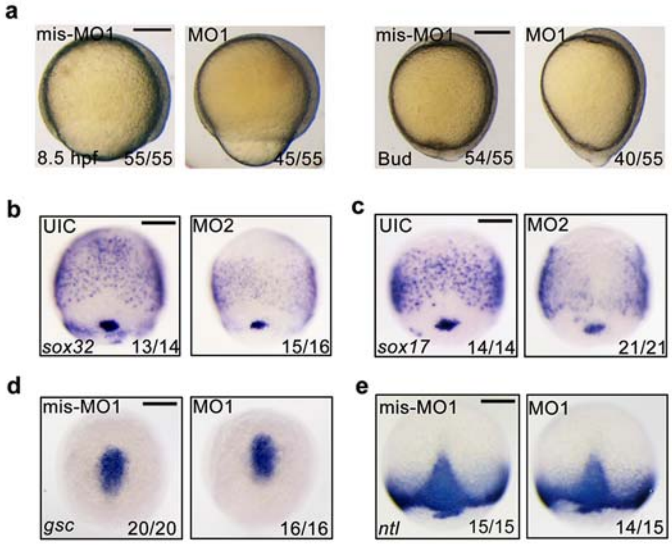

Supplementary Figure 3. *fscn1a* morphants exhibit defects in epiboly progression and endoderm formation. (a) Representative bright-field images of 4 ng *fscn1a* mis-MO1 and MO1 injected embryos at 8.5 hpf and bud stages. Lateral views with dorsal to the right. (b-c) Knockdown of *fscn1a* by injection of 25 ng *fscn1a* MO2 dramatically reduced the expression of the early endodermal markers *sox32* (b) and *sox17* (c) at the 75% epiboly stage. Panels are shown in dorsal view with anterior to the top. (d-e) The expression of the mesendodermal markers is normal in *fscn1a* morphants. Embryos injected with mis-MO1 (4 ng) or *fscn1a* MO1 (4 ng) at the one-cell stage and harvested at the 75%-epiboly stage for *in situ* hybridization with *gsc* (d) and *ntl* (e) probes. Dorsal views with anterior to the top. Scale bar, 200 μm.

Supplementary Figure 4

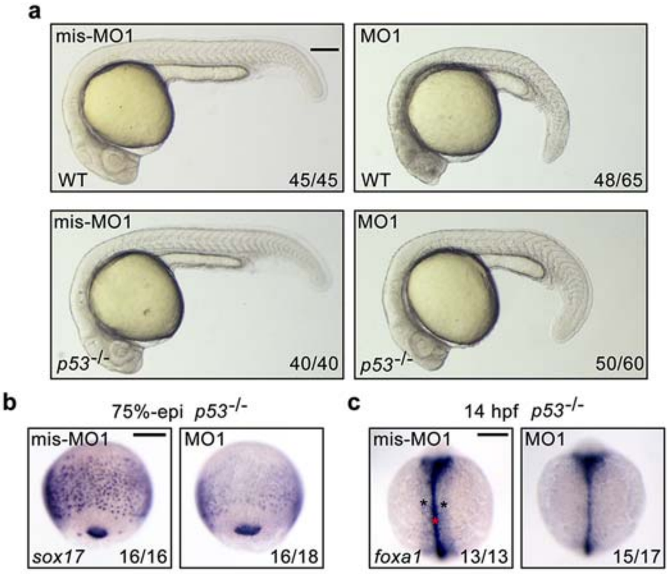

Supplementary Figure 4. *fscn1a* knockdown in *p53$^{-/-}$* mutants induce obvious defects in endoderm formation. (a) Representative bright-field images of wild-type and *p53$^{-/-}$* mutant embryos injected with 4 ng *fscn1a* mis-MO1 or MO1 at 24 hpf. Lateral views with dorsal to the right. Scale bar, 200 μm. (b-c) The expression of endodermal markers in *p53$^{-/-}$* mutant embryos at 75%-epiboly stage (b) and 14 hpf (c). (b) Lateral views with dorsal to the right. Scale bar, 200 μm.

Supplementary Figure 5

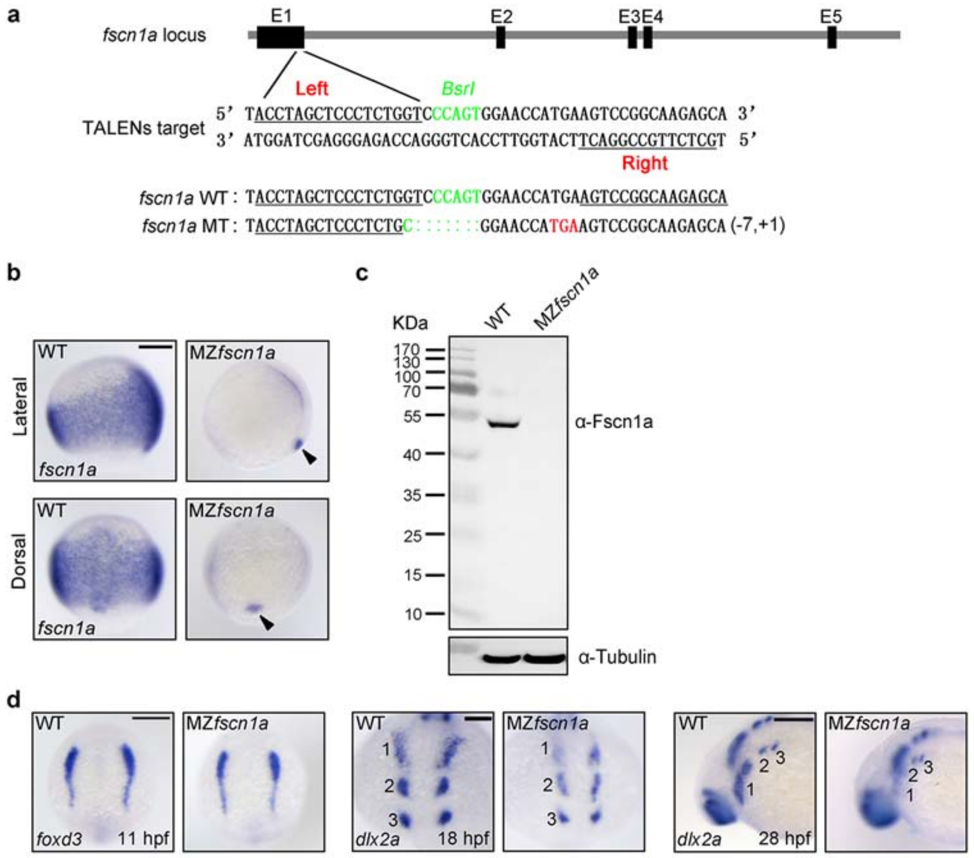

Supplementary Figure 5. MZ*fscn1a* mutants display migration defects in the first NC stream. (a) Generation of *fscn1a* mutant with TALENs. The “Left” and “Right” binding sites for TALENs were underlined and the *BSRI* site was shown in green used for detection of mutations by restriction enzyme digestion. One mutation which led to a shift of the open reading frame with a premature stop codon (red) was identified from the F_1 embryos of founder fish. (b-c) The expression of *fscn1a* mRNA and protein in wild-type and MZ*fscn1a* mutant embryos. Wild-type and MZ*fscn1a* mutant embryos were harvested at the 75%-epiboly stage for *in situ* hybridization (b) and immunoblotting (c). Note that the forerunner cell expression of *fscn1a* transcripts is not changed in MZ*fscn1a* mutants (b, indicated by arrow head). Scale bar, 200 µm. (d) Wild-type and MZ*fscn1a* mutant embryos were harvested at 11 hpf (Scale bar, 200

μm), 18 hpf (Scale bar, 100 μm) and 28 hpf (Scale bar, 200 μm) for *in situ* hybridization with *foxd3* and *dlx2a* probes. Note that MZ*fscn1a* mutants have normal NC induction but decreased number of *dlx2a*-expressing NC cells in the first stream.

Supplementary Figure 6

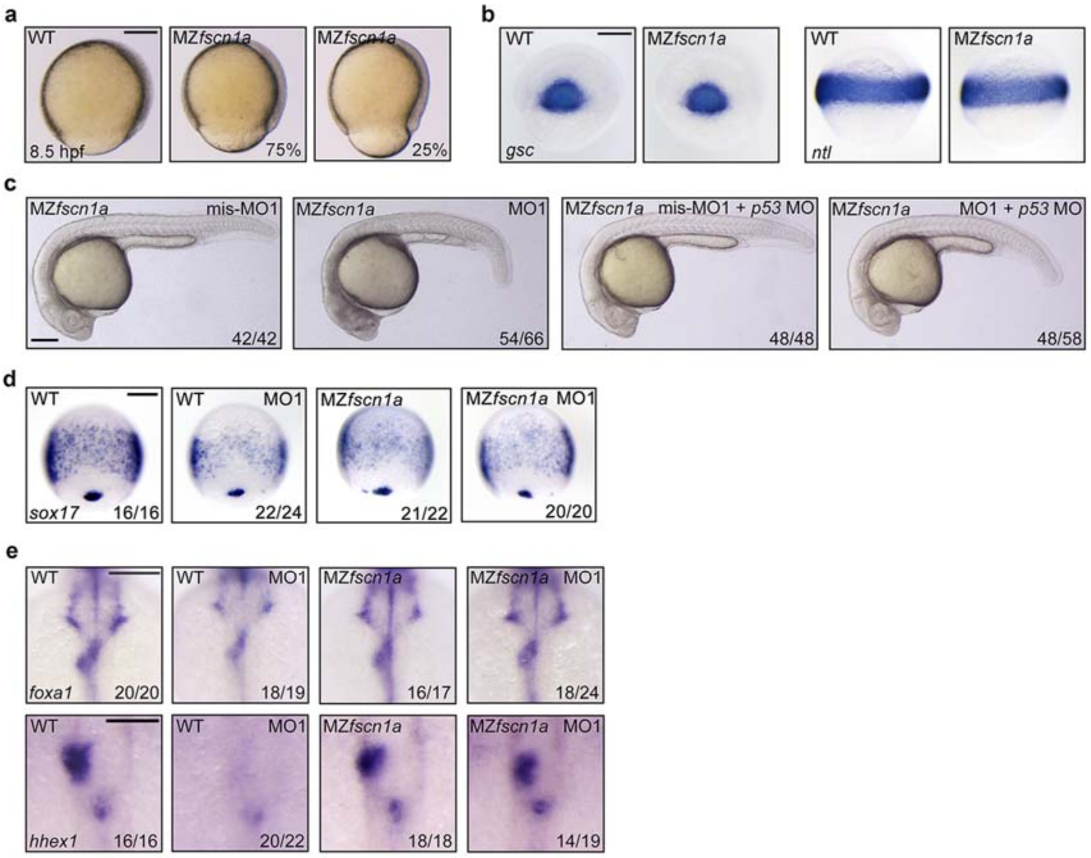

Supplementary Figure 6. MZ*fscn1a* mutants exhibit defects in epiboly progression and endoderm development, but have normal mesoderm formation. (a) Representative bright-field images of wild-type and MZ*fscn1a* mutants at 8.5 hpf. The percentage of MZ*fscn1a* mutants with various degrees of epiboly defects was shown. Scale bar, 200 μm. (b) Wild-type and MZ*fscn1a* mutant embryos were harvested at the shield stage for *in situ* hybridization with *gsc* and *ntl* probes. Scale bar, 200 μm. (c) Representative bright-field images of MZ*fscn1a* mutant embryos injected with indicated 4 ng *fscn1a* MOs together with or without 4 ng *p53* MO at 24 hpf. Scale bar, 200 μm. (d) The expression of *sox17* at 75% epiboly stage was examined in wild-type and MZ*fscn1a* mutant embryos injected with indicated *fscn1a* MOs. Note that no visible enhancement of endodermal related defects was observed

in MO1 injected MZ*fscn1a* embryos compared with uninjected mutants. Scale bar, 200 µm. (e) The expression of *foxa1* and *hhex1* at 28 hpf was examined in wild-type and MZ*fscn1a* mutant embryos injected with indicated *fscn1a* MOs. Note that the liver and pancreatic buds were normally formed in MO1 injected MZ*fscn1a* embryos compared with uninjected mutants. Scale bar, 200 µm in the upper panels and 100 µm in the lower panels.

Supplementary Figure 7

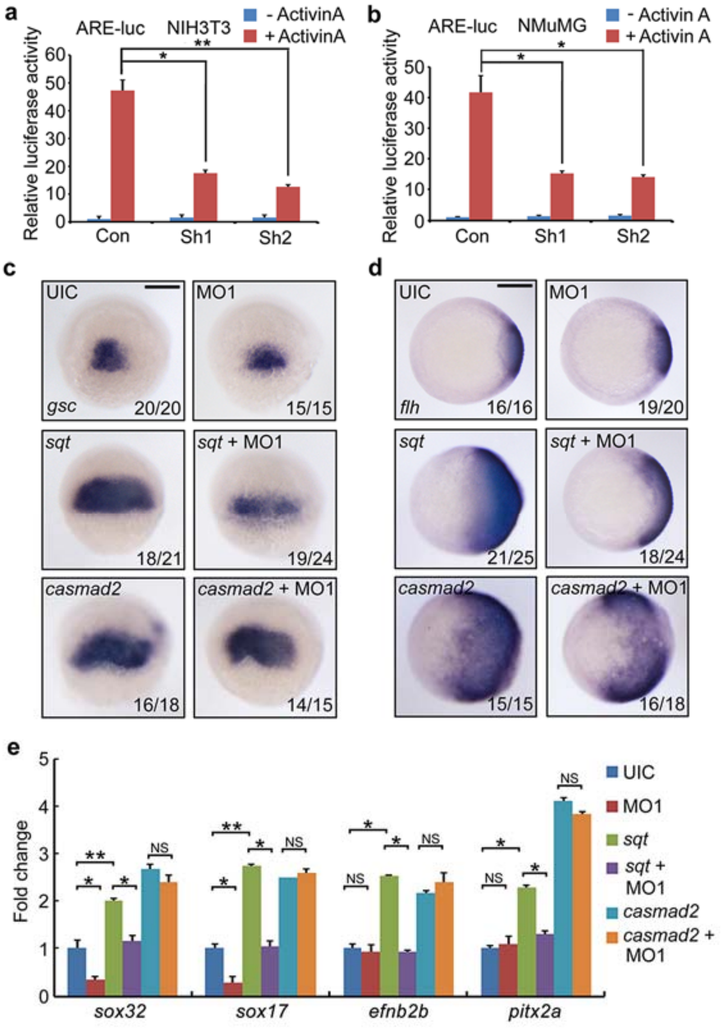

Supplementary Figure 7. Knockdown of Fscn1a impairs *sqt* ectopic expression-induced exression of target genes. (a-b) mammalian Fscn1 is required for Activin signal transduction. NIH3T3 (a) and NMuMG (b) cells transfected with the indicated plasmids (2 μg shRNA plasmids together with or without 0.5 μg reporter plasmids in one well of 6-well plate) were treated with Activin A (25 ng/ml) for 12 hours before harvested for luciferase measurements. The relative luciferase activity was the mean with SD from three independent experiments. Student's t test, *$P < 0.05$, **$P < 0.01$. (c-e) *fscn1a* is essential for Nodal signal to regulate target gene

expression. Wild-type embryos were injected with indicated MOs and mRNAs and harvested at shield stage for *in situ* hybridization (c and d) and real-time PCR experiments (e). Note that the *sqt o*verexpression induced- but not *casmad2 o*verexpression induced-expression of Nodal signal target genes was inhibited by *fscn1a* MO1 injection. Scale bar, 200 μm. In panel e, the expression of *β-actin* was used as a reference to normalize the amount of mRNAs in each sample. The data are presented as mean ±SD of three independent experiments. Student's t test, **P* < 0.05, ***P* < 0.01; NS, nonsignificant. UIC, uninjected control.

Supplementary Figure 8

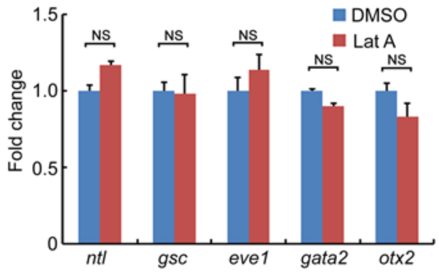

Supplementary Figure 8. Lat A treatment does not influence the mesoderm and ectoderm formation. The expression of mesodermal markers (*ntl*, *gsc* and *eve1*), non-neural ectodermal marker (*gata2*) and neuroectodermal marker (*otx2*) was assessed by real-time PCR at the shield stage in zebrafish embryos treated with 0.1 μM Lat A from the sphere stage. The expression of *β-actin* was used as a reference to normalize the amount of mRNAs in each sample. The data are presented as mean ±SD of three independent experiments. Student's t test, NS, nonsignificant.

Supplementary Figure 9

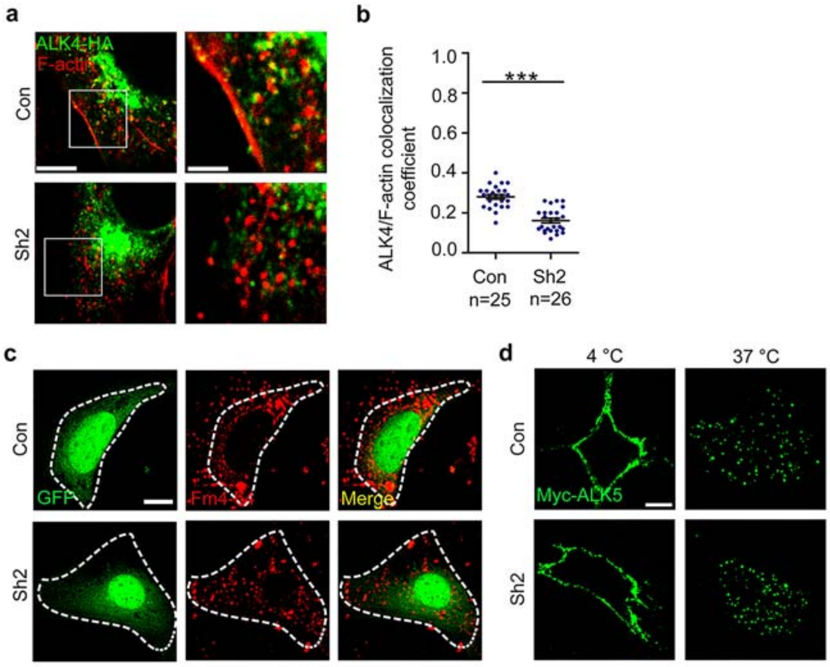

Supplementary Figure 9. Fscn1-deletion interferes with the co-localization of ALK4 and F-actin, but does not affect the internalization of type I receptors. (a-b) NIH3T3 cells cotransfected with ALK4-HA and shRNA plasmids were fixed and costained with phalloidin-TRITC and anti-HA antibody to show the colocalization of F-actin (red) and ALK4 (green). The boxed area in the left image (Scale bar, 5 μm) is presented at a higher magnification in the corresponding right image (Scale bar, 2 μm) (a). Peason's colocalization coefficient was quantified from the indicated cell numbers in three independent experiments and the group values are expressed as mean±SD. Student's t test, ***$P < 0.001$ (b). (c) Representative fluorescence micrographs from experiments examining the uptake of FM4-64-labeled plasma membrane in NIH3T3 cells. NIH3T3 cells transfected with the indicated shRNA plasmids with a GFP marker were incubated with FM4-64 for 1 hour, fixed, and imaged using Nikon A1R+ confocal microscope system. Scale bar, 5 μm. (d) NIH3T3 cells expressing

Myc-tagged ALK5 were incubated with mouse anti-Myc antibody at 4 °C for 5 h then incubated with PBS containing 10% FBS at 37 °C for 30 min. The cells were visualized by immunofluorescence with anti-Myc (green). Scale bar, 5 μm. In the colocalization analysis, NIH3T3 cells were cultured in 6-well plates. The dose of transfected plasmid DNA: Myc-ALK5, 1 μg; ALK4-GFP, 0.5 μg.

Supplementary Figure 10 (1 of 4)

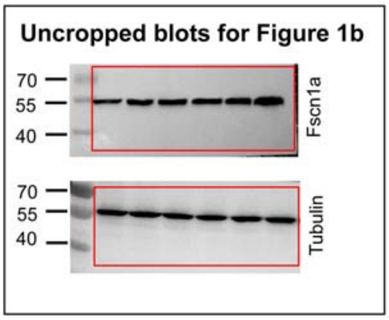

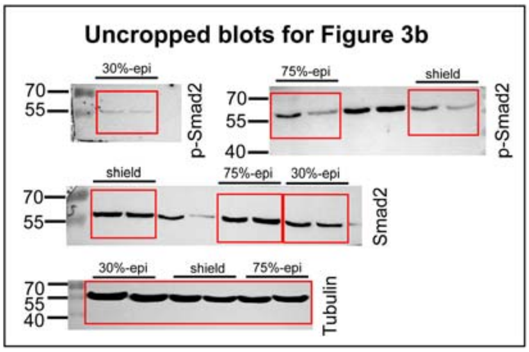

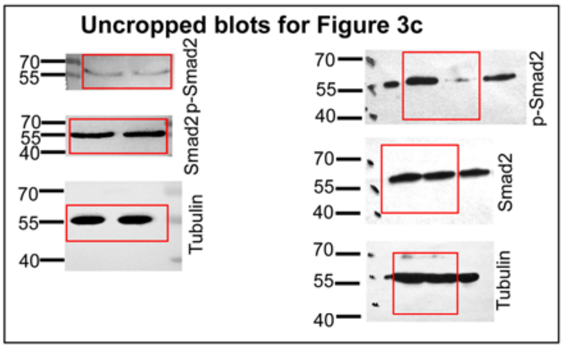

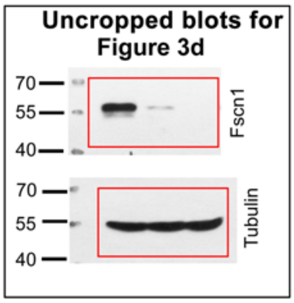

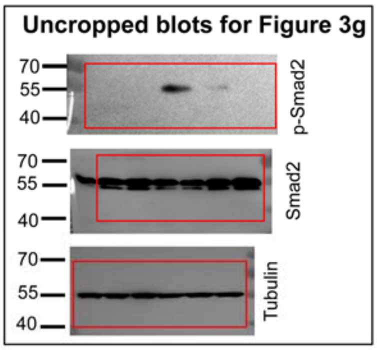

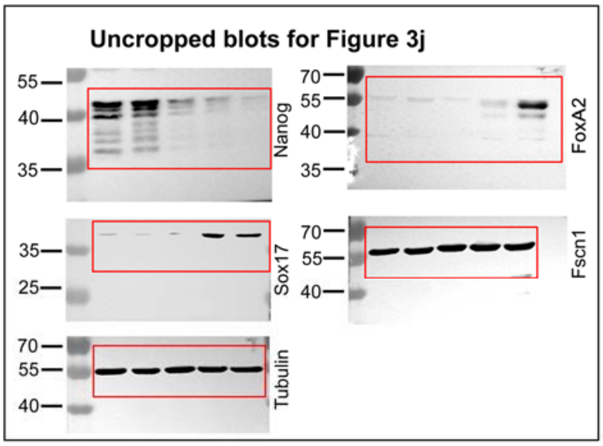

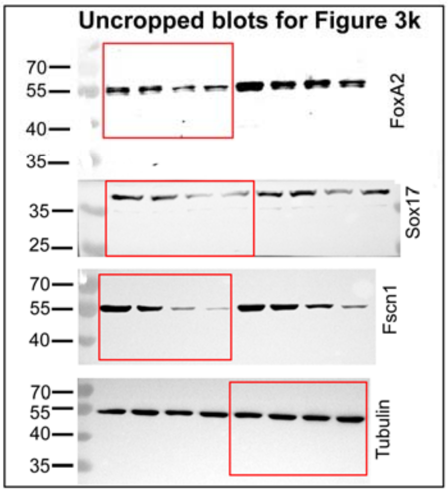

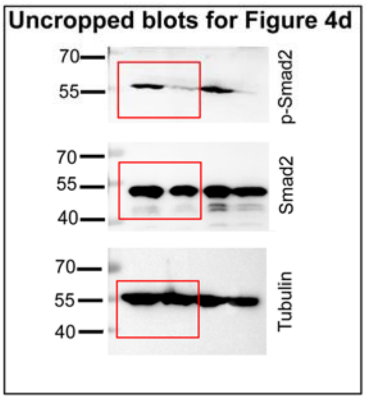

Supplementary Figure 10 (2 of 4)

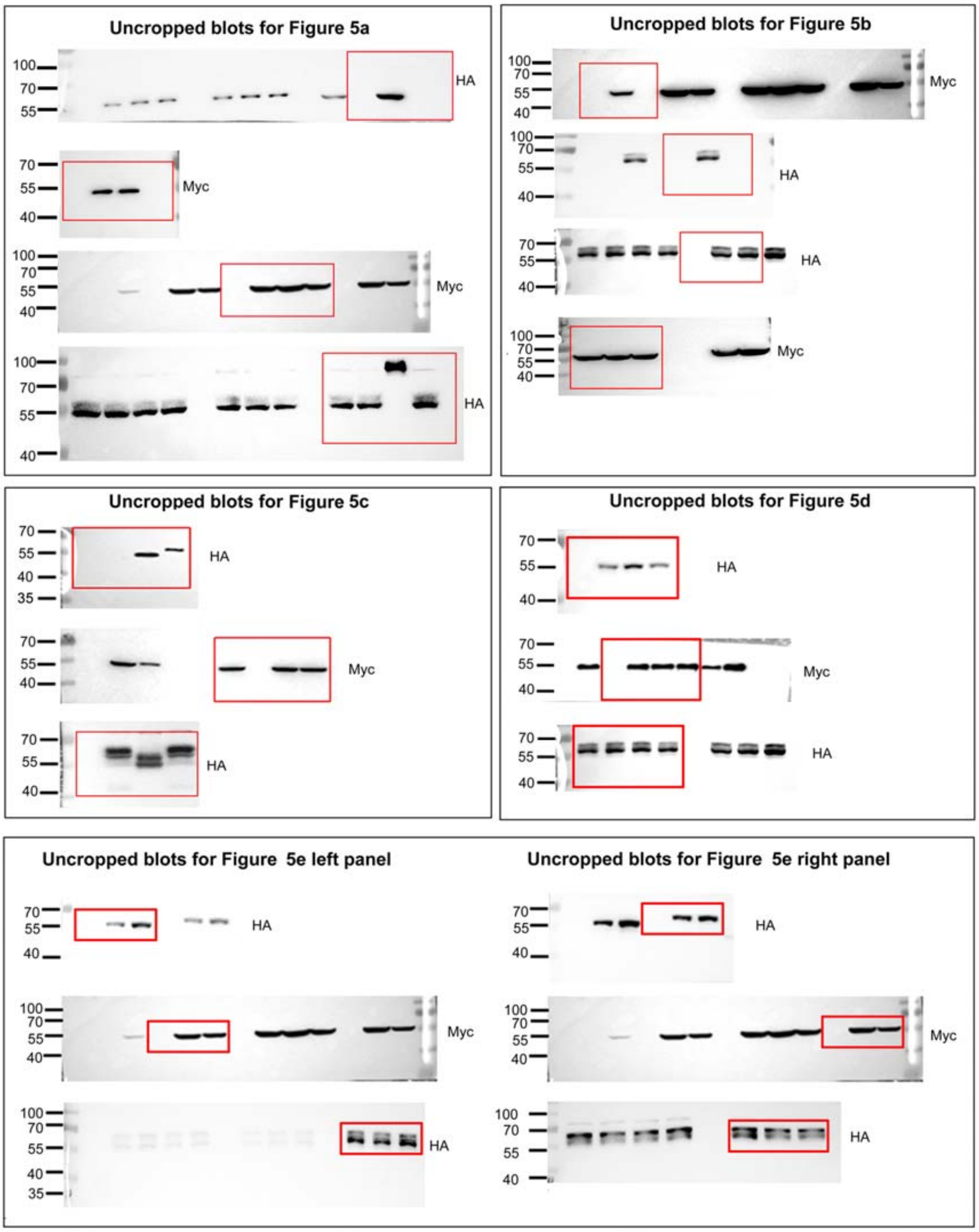

Supplementary Figure 10 (3 of 4)

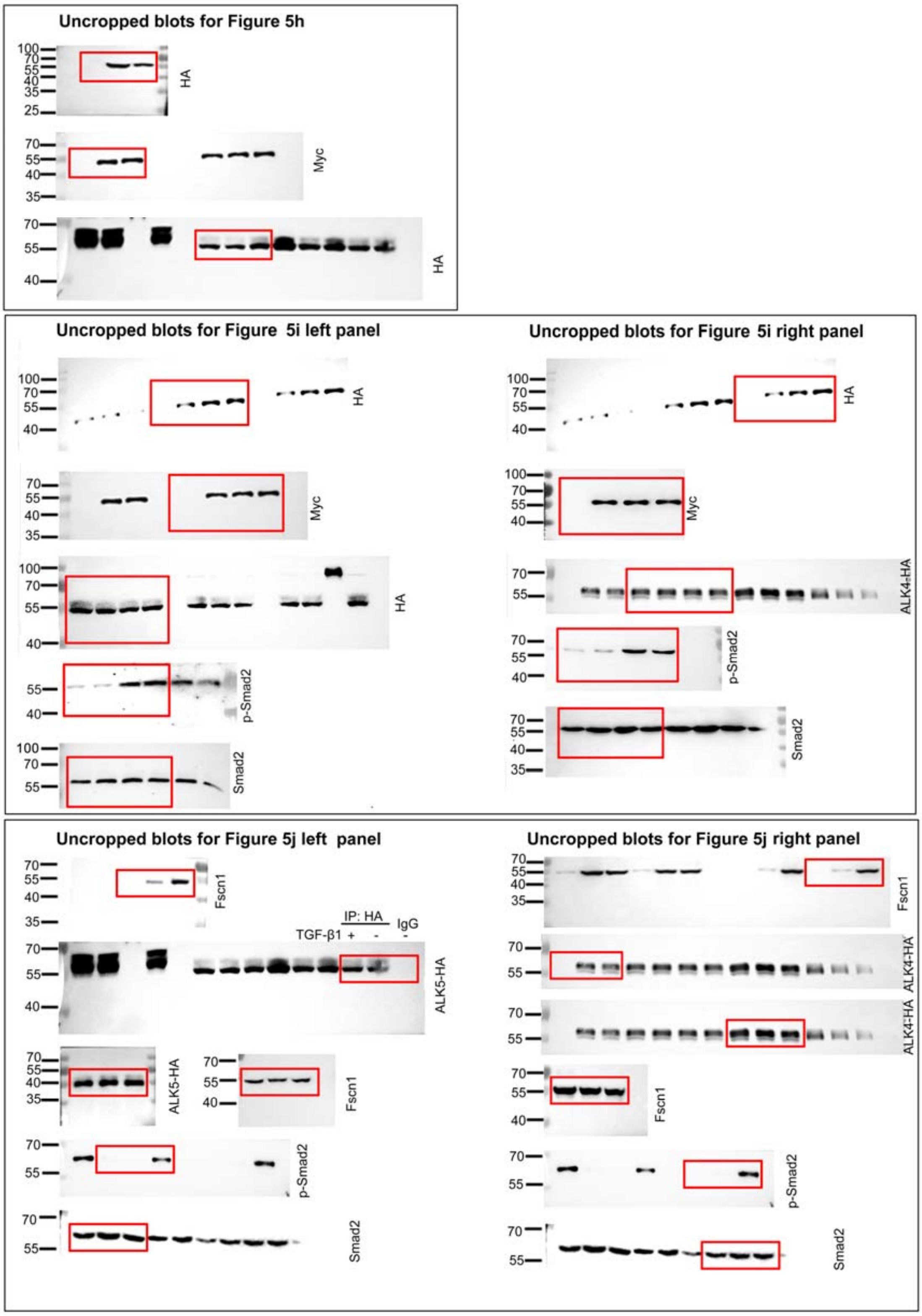

Supplementary Figure 10 (4 of 4)

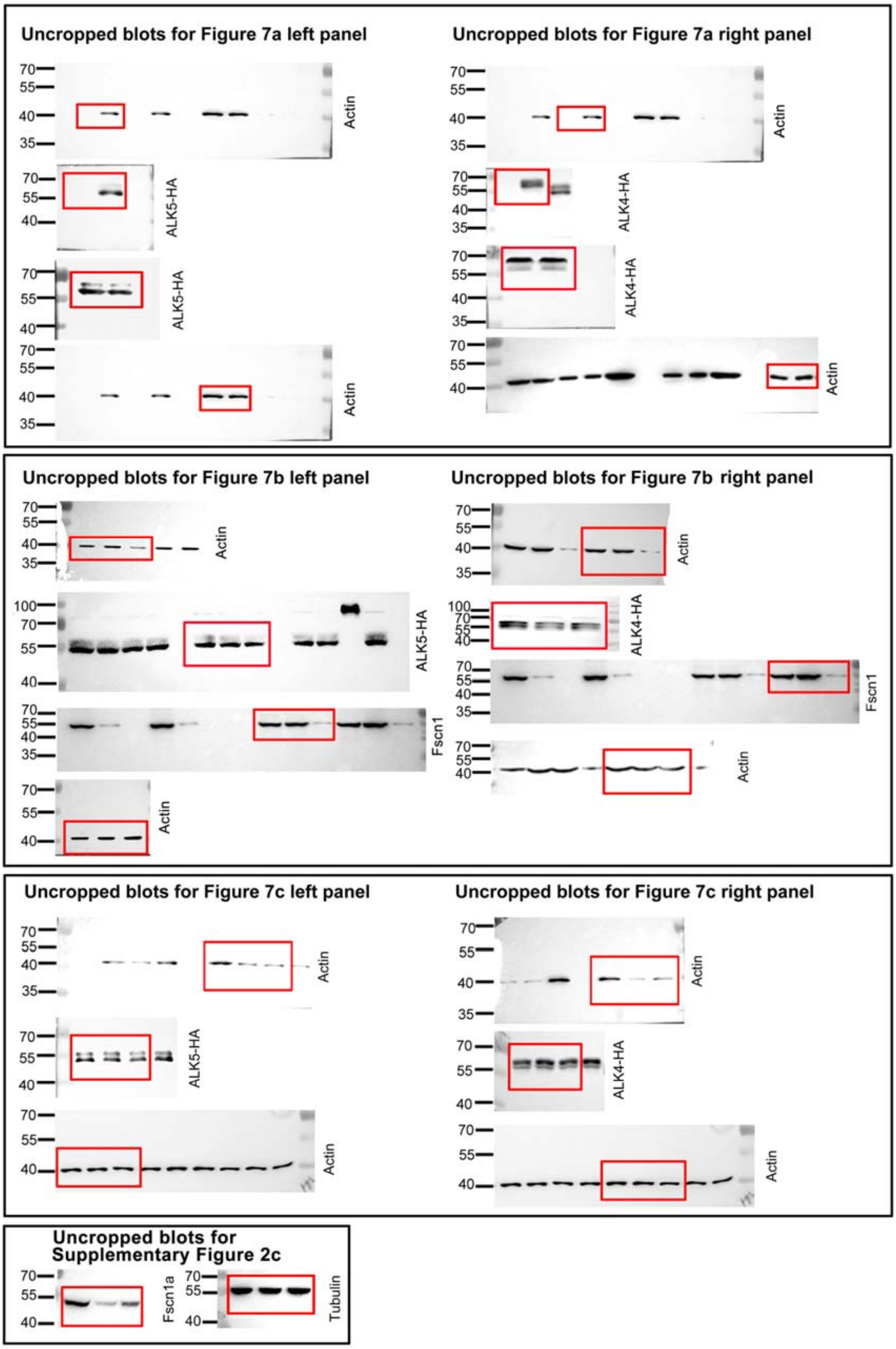

Supplement: Supplementary Information — Supplementary Figures 1-10 [file ncomms12603-s1.pdf]
